# Supplementary material for: Ion pair sites for efficient electrochemical extraction of uranium in real nuclear wastewater
Source: Nat Commun. 2024 May 16;15:4149. doi: 10.1038/s41467-024-48564-y (PMC11099191; doi:10.1038/s41467-024-48564-y)
Supplement: Supplementary file 1 — Supplementary Information [file 41467_2024_48564_MOESM1_ESM.pdf]

Supplementary Information

Ion pair sites for efficient electrochemical extraction of uranium in real nuclear wastewater

Tao Lin[1], Tao Chen[1], Chi Jiao[2], Haoyu Zhang[1], Kai Hou[1], Hongxiang Jin[1], Yan Liu[2,*], Wenkun Zhu[1,*] & Rong He[1,*]

[1] State Key Laboratory of Environment-friendly Energy Materials, School of Life Science & Engineering, School of Materials & Chemistry, National Co-innovation Center for Nuclear Waste Disposal & Environmental Safety, Sichuan Civil-military Integration Institute, Southwest University of Science & Technology, Mianyang 621010, P. R. China. [2] School of Chemistry and Materials Science, Anhui Normal University, Wuhu 241002, P. R. China.

Correspondence and requests for materials should be addressed to Yan Liu (email: ly0201@ahnu.edu.cn, ORCID: 0000-0001-6945-9942), Wenkun Zhu (email: zhuwenkun@swust.edu.cn, ORCID: 0000-0002-4116-6213) & Rong He (email: her@swust.edu.cn, ORCID: 0000-0002-4023-5934).

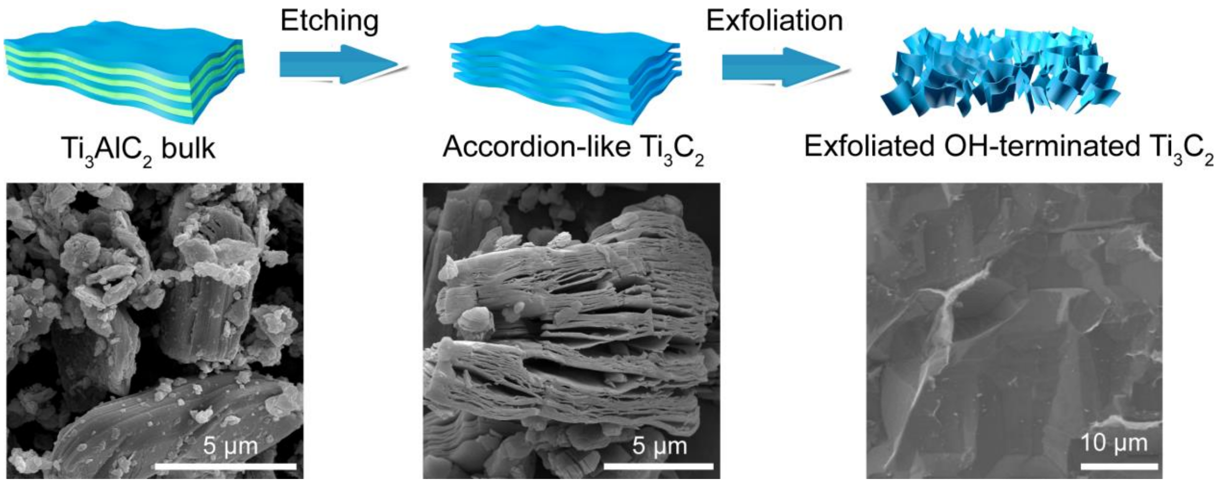

Supplementary Fig. 1 The synthesis schematic diagram of OH-terminated Ti_3C_2 nanosheets. Initially, the Al layers of bulk Ti_3AlC_2 were etched to form the accordion-like Ti_3C_2 nanosheets. Followed by the exfoliation process under N_2 protection in aqueous solution, the accordion-like Ti_3C_2 nanosheets were transformed into lamellar OH-terminated Ti_3C_2 nanosheets. Credits: Copyright (top, schematic models) Hangzhou SPHERE Technology Co., Ltd. Source data are provided as a Source Data file.

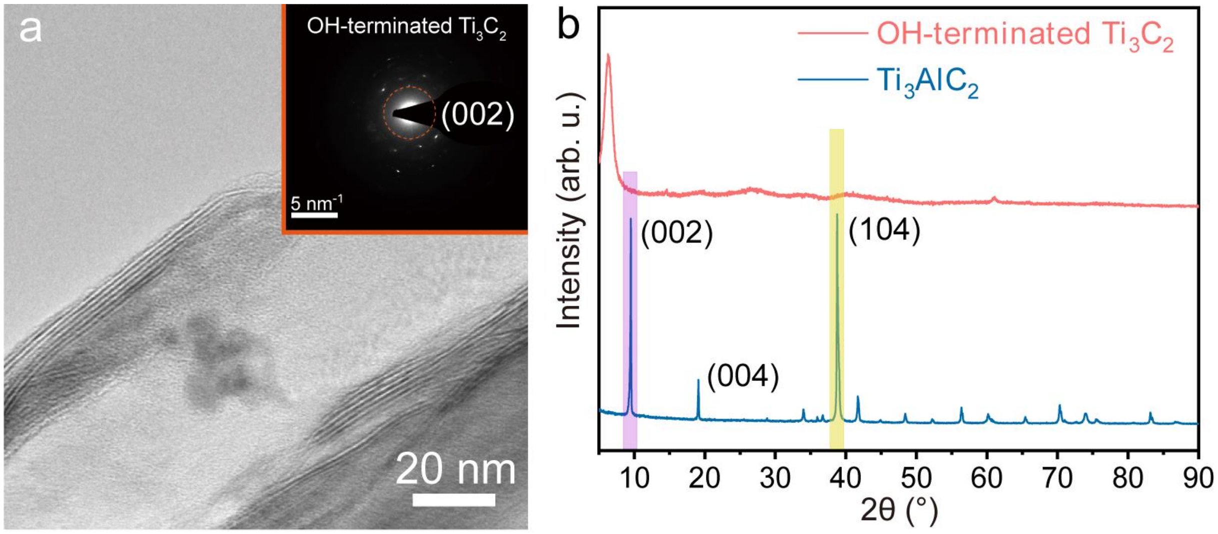

Supplementary Fig. 2 The structure of lamellar OH-terminated Ti_3C_2. a HRTEM and SAED image of OH-terminated Ti_3C_2 nanosheets. The inset is the SAED pattern of the OH-terminated Ti_3C_2. **b** The XRD patterns of bulk Ti_3AlC_2 and exfoliated OH-terminated Ti_3C_2 nanosheets. The highlighted regions display the characteristic peaks of the interlayer spacing (002) in Ti_3AlC_2 and the elemental Al (104). Source data are provided as a Source Data file.

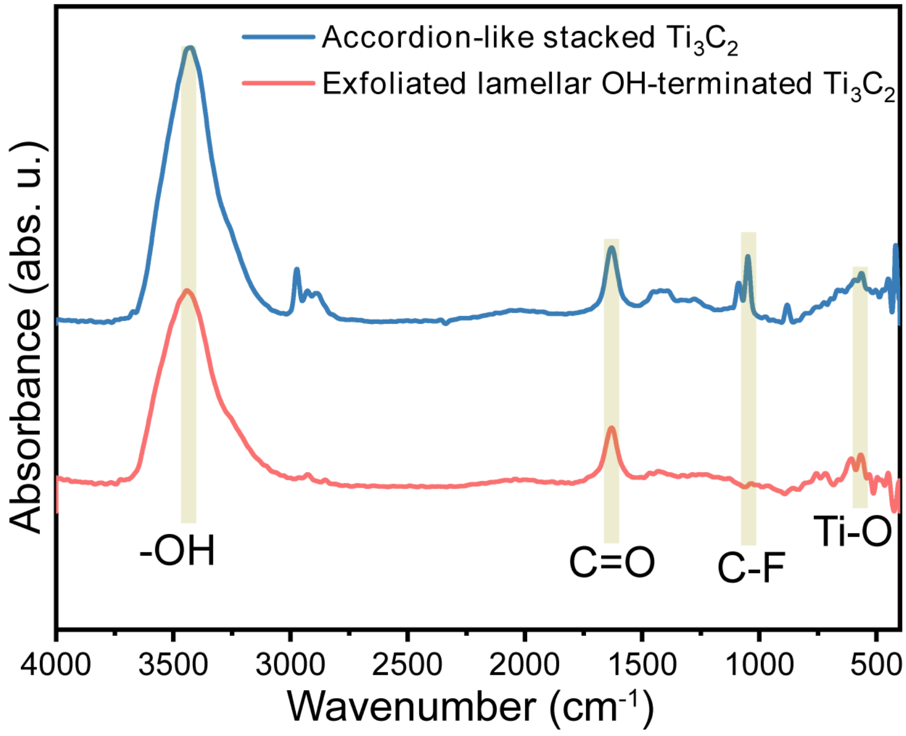

Supplementary Fig. 3 The change of surface functional groups. FTIR spectra of as-synthesized accordion-like stacked Ti_3C_2 nanosheets and exfoliated lamellar OH-terminated Ti_3C_2 nanosheets. The highlighted regions display the characteristic peaks of -OH, C=O, C-F, and Ti-O bonds, respectively[1,2]. Source data are provided as a Source Data file.

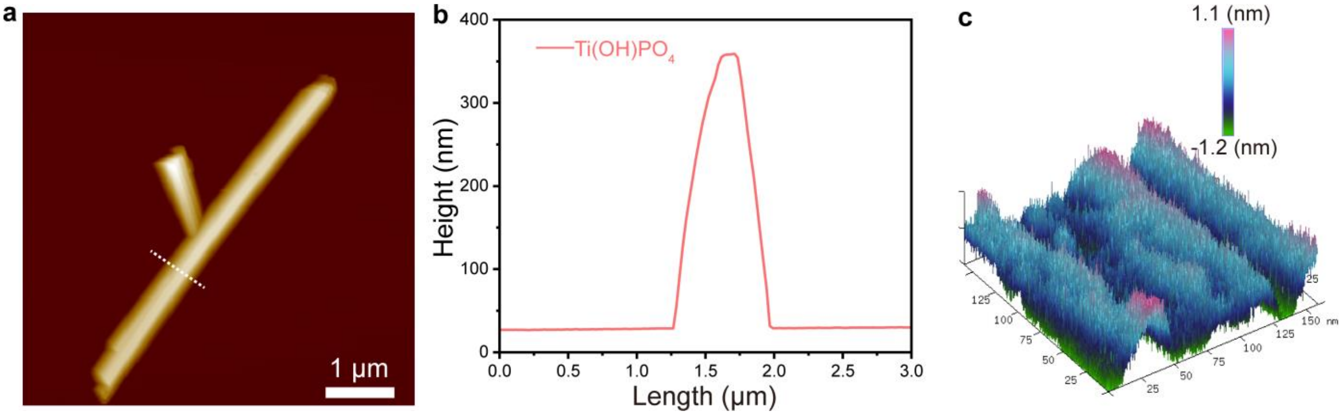

Supplementary Fig. 4 The morphology structure of $Ti(OH)PO_4$ nanorods. a AFM images of $Ti(OH)PO_4$ nanorods. **b** Corresponding height profile of $Ti(OH)PO_4$ nanorods. **c** Corresponding three-dimensional graph of $Ti(OH)PO_4$ nanorods. Source data are provided as a Source Data file.

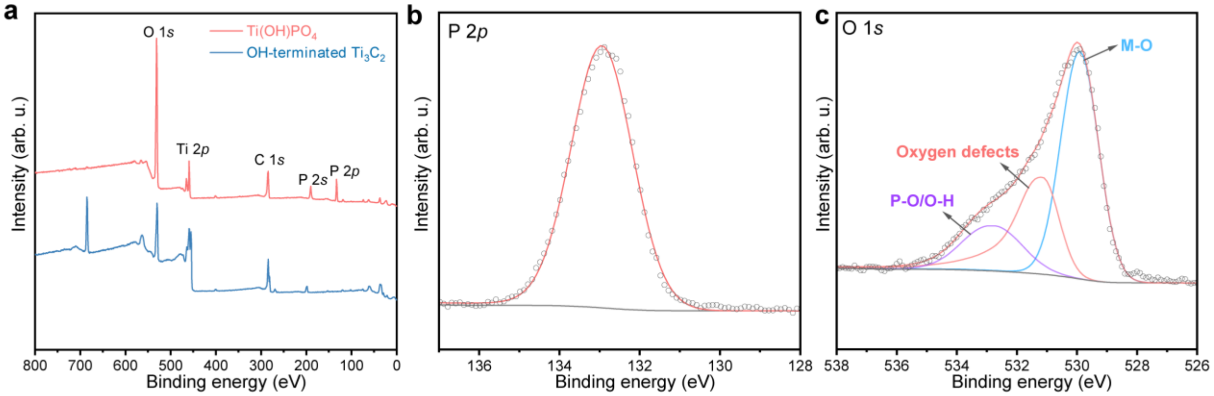

Supplementary Fig. 5 The surface phosphate groups of $Ti(OH)PO_4$. a The XPS spectrum of $Ti(OH)PO_4$ and OH-terminated Ti_3C_2 nanosheets. **b** P 2*p* and **c** O 1*s* spectrum of $Ti(OH)PO_4$ nanorods. Source data are provided as a Source Data file.

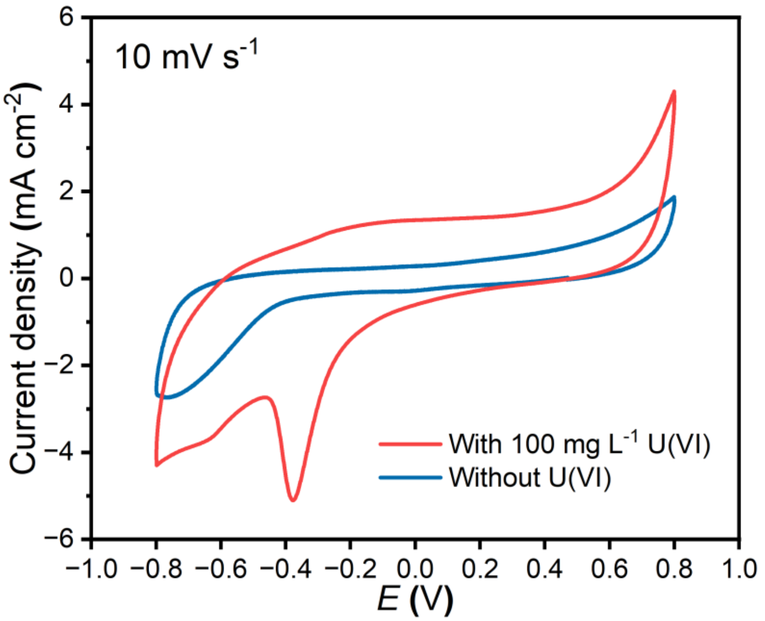

Supplementary Fig. 6 The origin of the reduction peak. The CV tests in the presence or absence of U(VI) in 30 g L^{-1} of F^{-} aqueous solution at a scan rate of 10 mV s^{-1}. *E* represents potential. Source data are provided as a Source Data file.

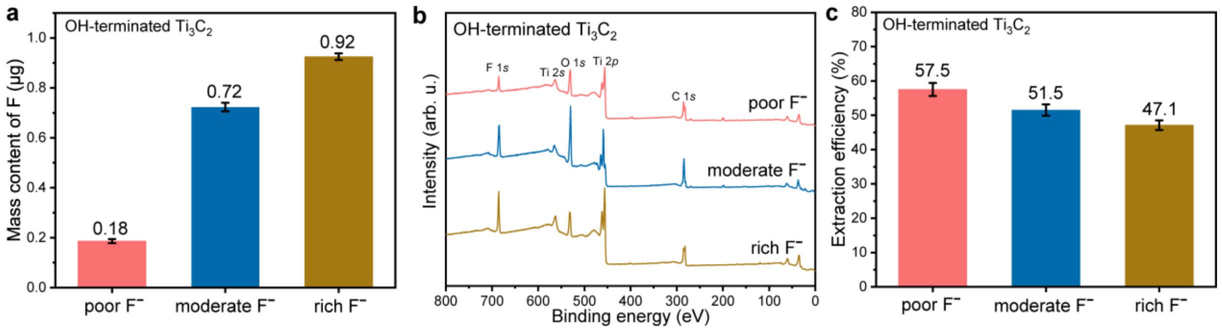

Supplementary Fig. 7 The relationship between surface F concentrations and uranium extraction performance of OH-terminated Ti_3C_2. a The mass content of F in the OH-terminated Ti_3C_2. **b** XPS spectrum of the OH-terminated Ti_3C_2 with different F contents. **c** The extraction efficiency of uranium in the OH-terminated Ti_3C_2. Error bars represent standard deviation of three measurements. Source data are provided as a Source Data file.

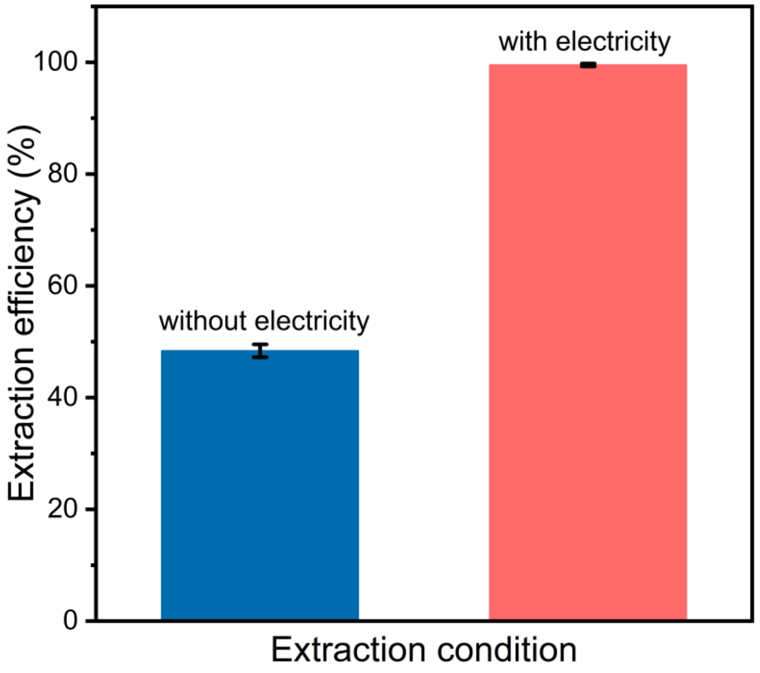

Supplementary Fig. 8 The extraction efficiency of uranium on $Ti(OH)PO_4$. The electrochemical extraction efficiency of U(VI) on $Ti(OH)PO_4$ nanorods exhibited a 2-fold enhancement. Error bars represent standard deviation of three measurements. Source data are provided as a Source Data file.

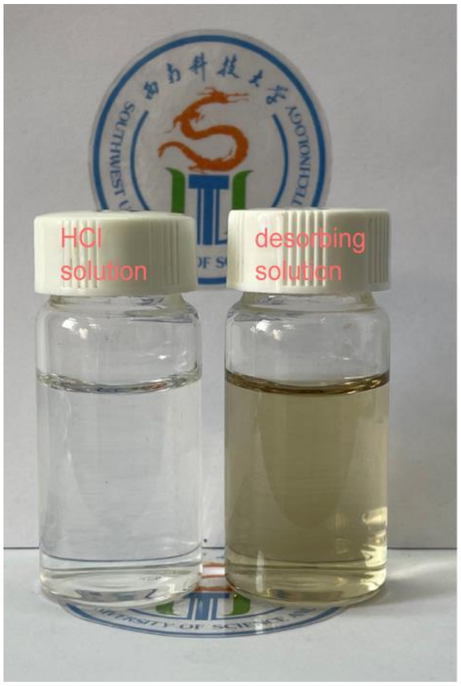

Supplementary Fig. 9 Desorption of electrodes. The color change of the HCl solution agent before and after desorption. Source data are provided as a Source Data file.

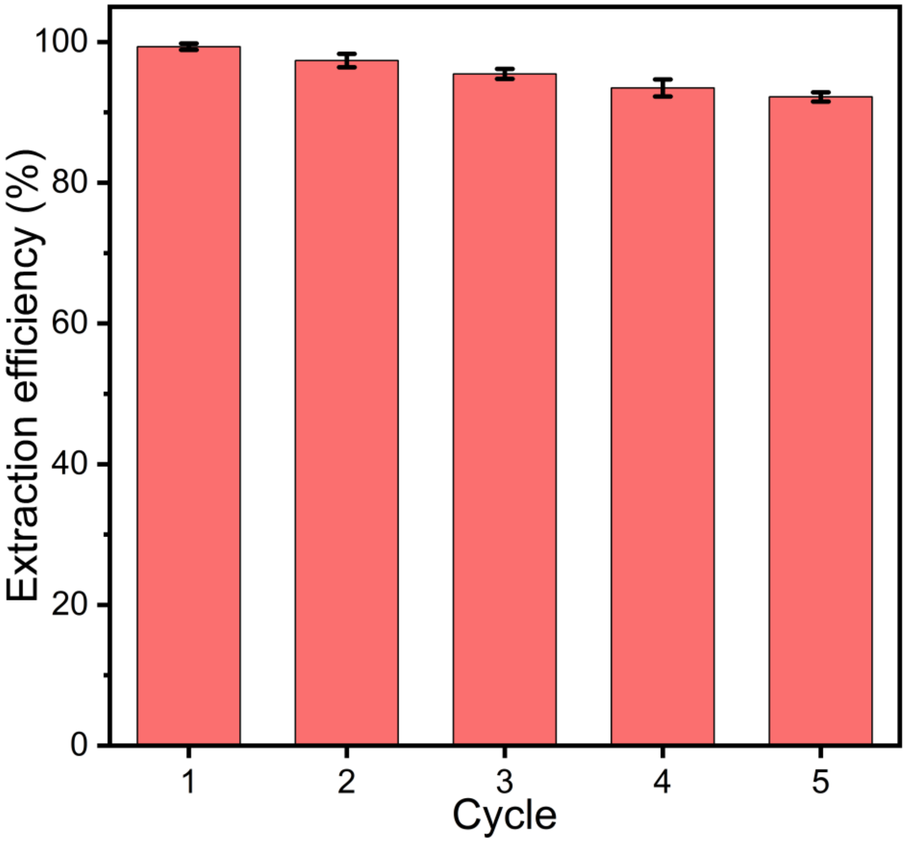

Supplementary Fig. 10 The cycle performance of Ti(OH)PO_4. The stability of electrochemical extraction of U(VI) on Ti(OH)PO_4 electrode throughout 5 electrocatalytic cycles. Error bars represent standard deviation of three measurements. Source data are provided as a Source Data file.

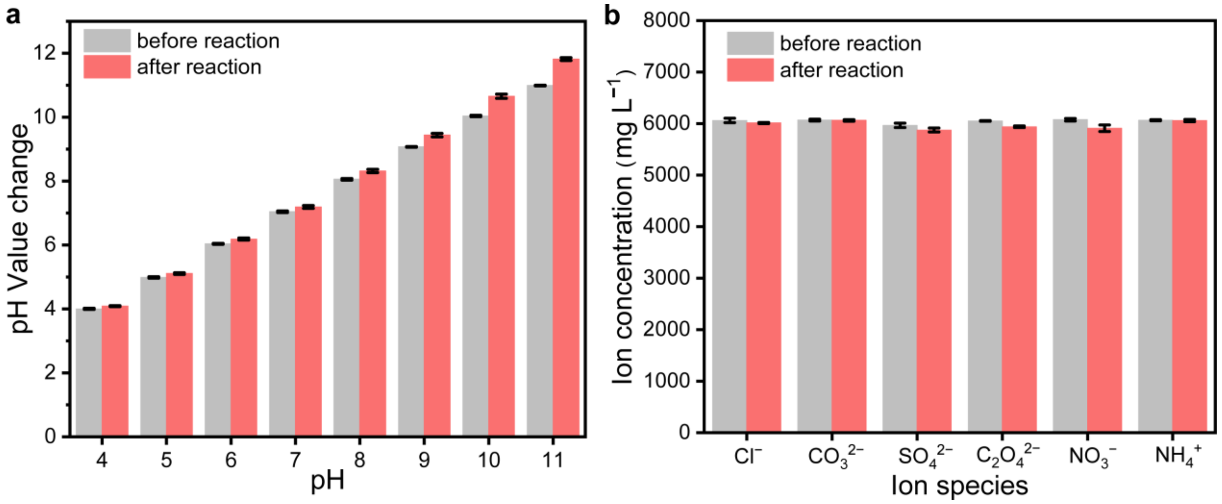

Supplementary Fig. 11 The changes in electrolytes during electrochemical processes. a pH value changes and **b** the concentration of other ions changes before and after the reaction. Error bars represent standard deviation of three measurements. Source data are provided as a Source Data file.

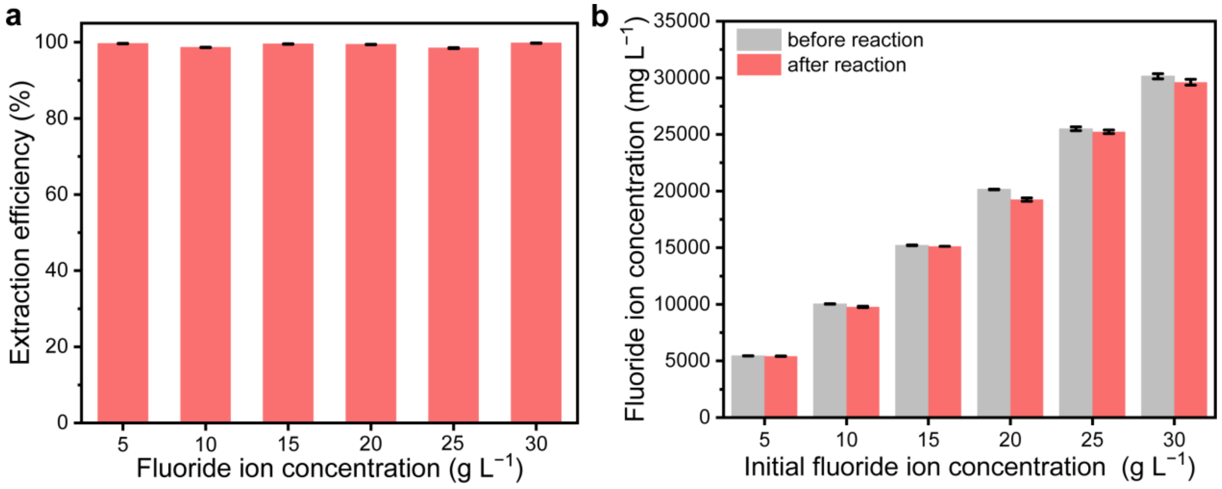

Supplementary Fig. 12 The electrochemical extraction performance of Ti(OH)PO_4. a The electrochemical extraction efficiency of U(VI) on Ti(OH)PO_4 under different concentrations of F^- varied from 5 g L^{-1} to 30 g L^{-1}. **b** The change of F^- concentrations before and after the reaction. Error bars represent standard deviation of three measurements. Source data are provided as a Source Data file.

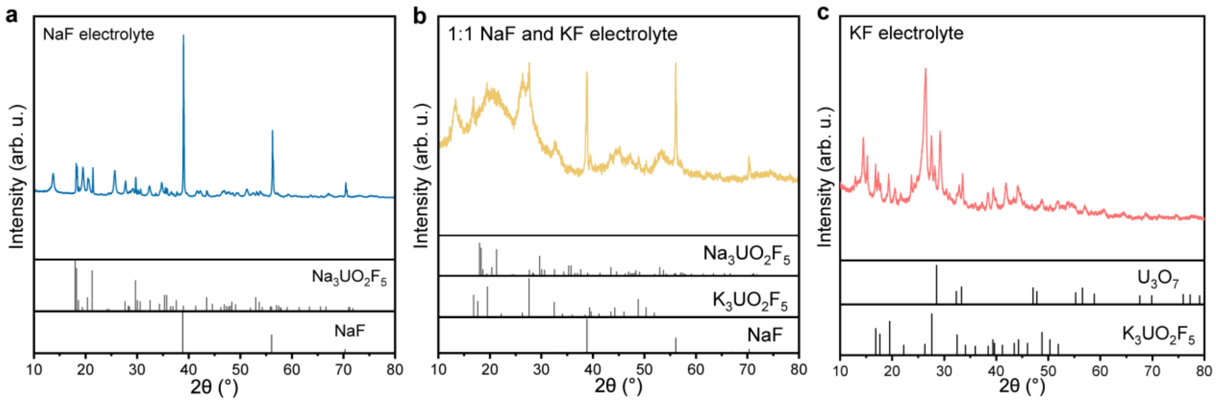

Supplementary Fig. 13 The influence of electrolytes on reduction products. **a** XRD pattern of the collected solid deposits at 30 g L^{-1} NaF electrolyte. **b** XRD pattern of the collected solid deposits at 15 g L^{-1} NaF and 15 g L^{-1} KF electrolyte. **c** XRD pattern of the collected solid deposits at 30 g L^{-1} KF electrolyte. Source data are provided as a Source Data file.

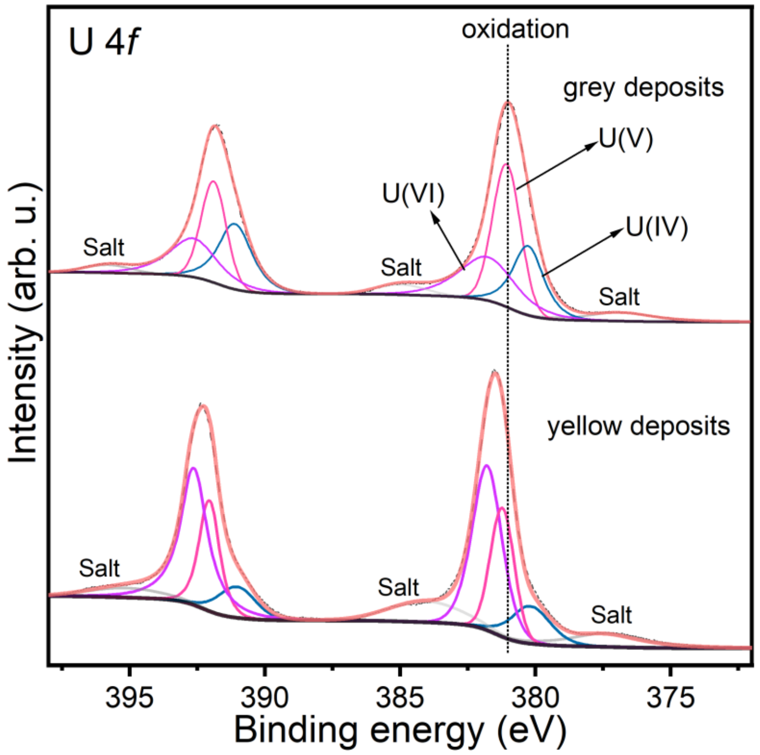

Supplementary Fig. 14 The valence state of uranium product. U 4*f* spectra of the grey and yellow deposits. The grey deposits possessed more content of U(IV) and U(V) than that of the yellow deposits. Source data are provided as a Source Data file.

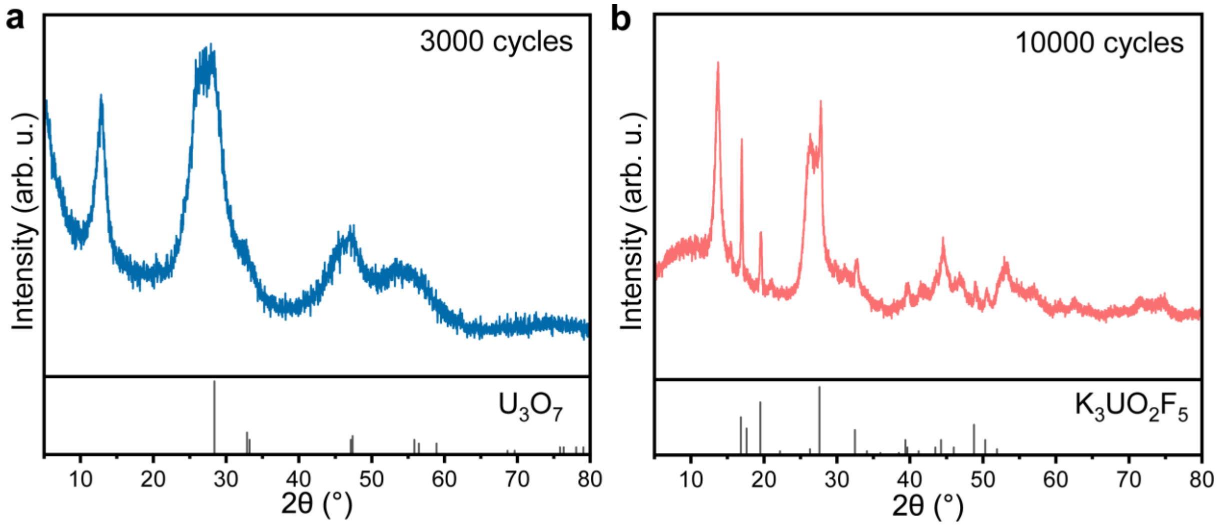

Supplementary Fig. 15 The structure of the deposits. a and **b** XRD pattern of the collected solid deposits at different CV cycles. Source data are provided as a Source Data file.

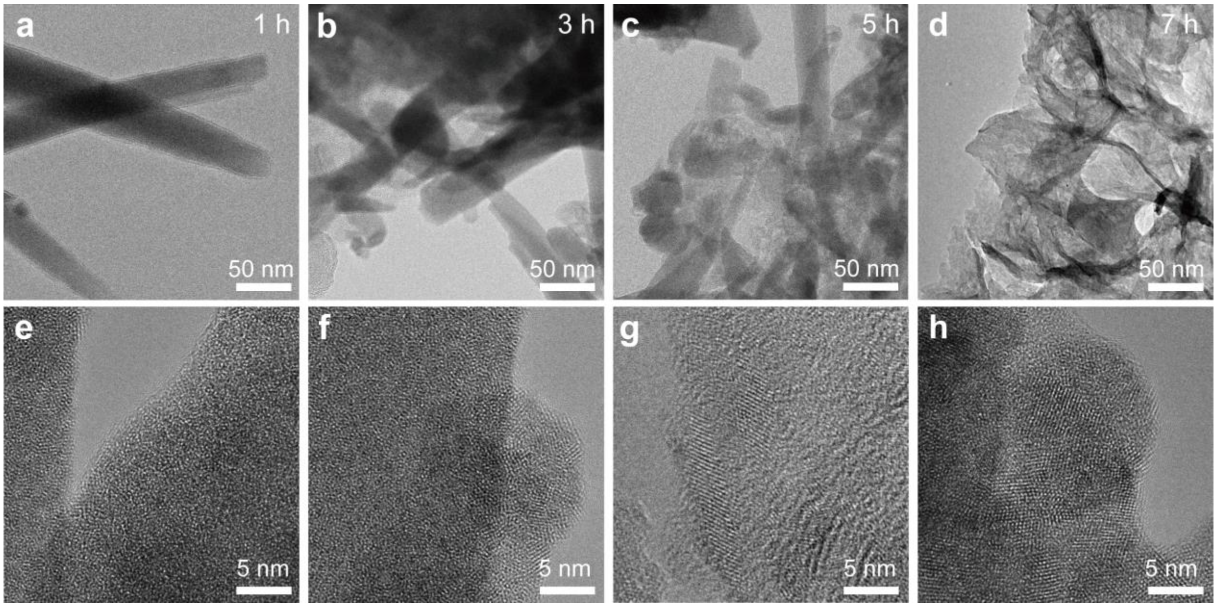

Supplementary Fig. 16 The alternation of the uranium species. a-d TEM and **e-h** HRTEM images of uranium species during the uranium extraction. Source data are provided as a Source Data file.

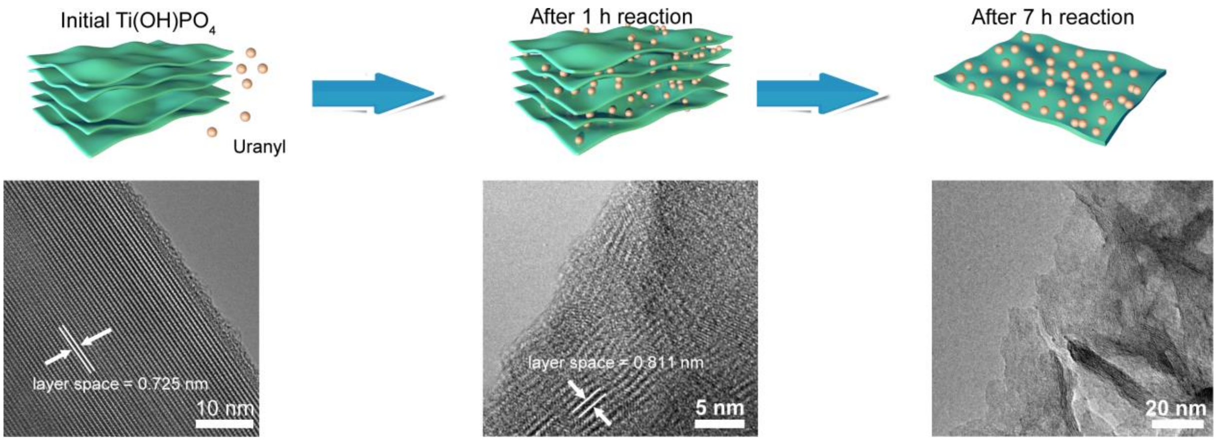

Supplementary Fig. 17 Morphological transformation of $Ti(OH)PO_4$. The schematic diagram of uranyl intercalation and the subsequent exfoliation of $Ti(OH)PO_4$ during the electrochemical uranium extraction process. Credits: Copyright (top, schematic models) Hangzhou SPHERE Technology Co., Ltd. Source data are provided as a Source Data file.

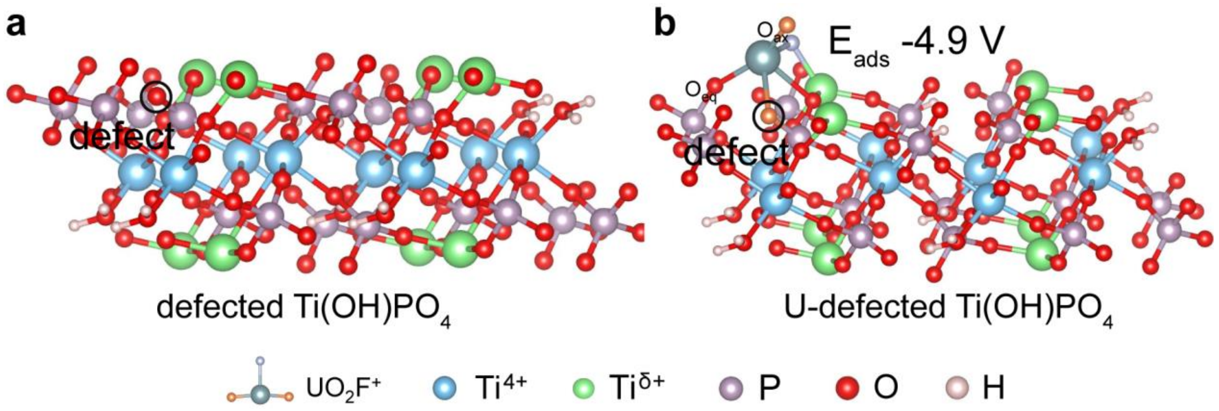

Supplementary Fig. 18 Reaction mechanism of defected Ti(OH)PO_4. Optimized adsorption configurations of UO_2F^+ adsorbed on the defected Ti(OH)PO_4. **a** The defected Ti(OH)PO_4. **b** The U-defected Ti(OH)PO_4. O_{ax}, the axial O of uranyl species; O_{eq}, the O atom at the adsorption site; E_{ads}, the adsorption energy of UO_2F^+. Source data are provided as a Source Data file.

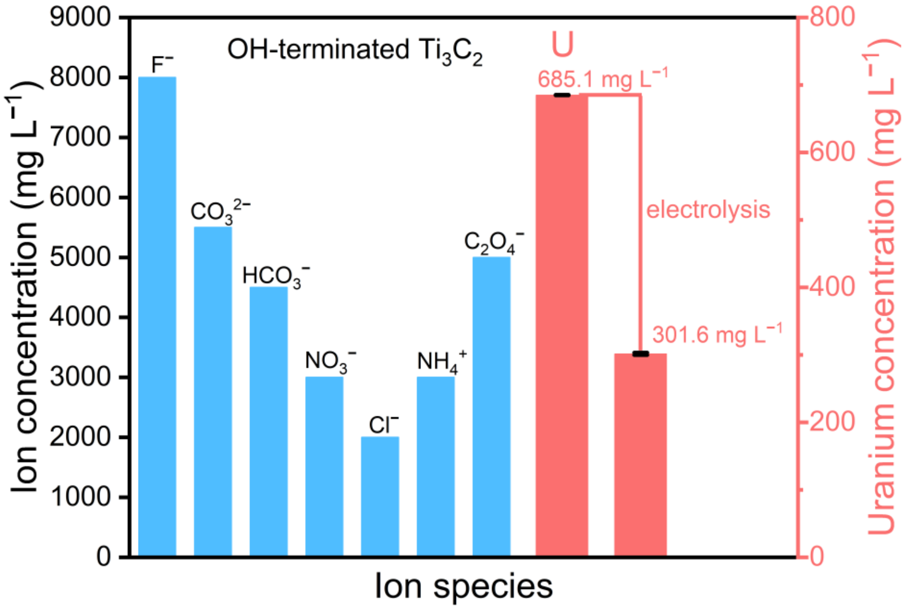

Supplementary Fig. 19 Experiment of real nuclear wastewater of OH-terminated Ti_3C_2. The electrochemical extraction efficiency of U(VI) on OH-terminated Ti_3C_2 in real nuclear wastewater. Error bars represent standard deviation of three measurements. Source data are provided as a Source Data file.

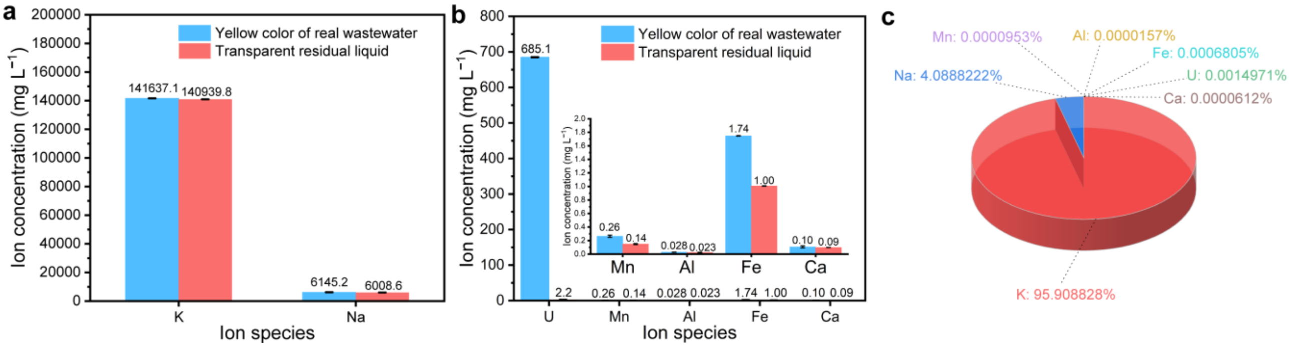

Supplementary Fig. 20 The concentration of all metal species before and after reaction. a and **b** The metal species concentrations of real wastewater and transparent residual liquid. Inset: the concentration changes of Mn, Al, Fe, and Ca. **c** The proportion of metal species of transparent residual liquid. Error bars represent standard deviation of three measurements. Source data are provided as a Source Data file.

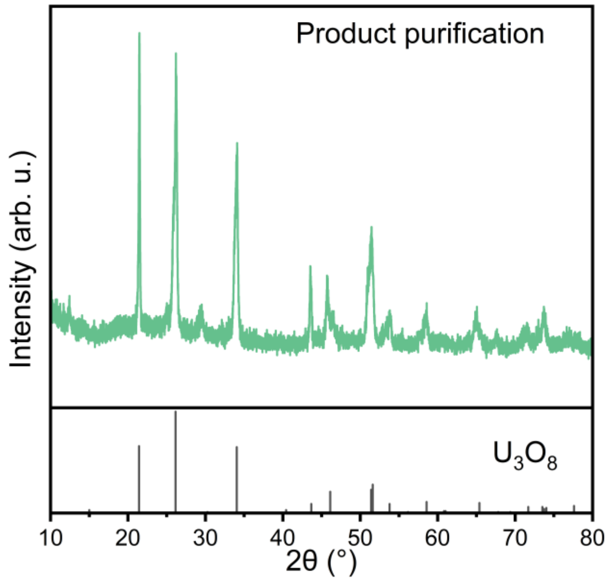

Supplementary Fig. 21 Analysis of uranium purification products. The XRD pattern of the purification product. The purified black-green uranium product was identified as U_3O_8 (JCPDS #31-1425). Source data are provided as a Source Data file.

Supplementary Table 1 The shift analysis of e_g and t_{2g} orbits in Ti L-edge XANES during the transformation of OH-terminated T_3C_2 into $Ti(OH)PO_4$.

Factor	e_g orbits	t_{2g} orbits
Coordination environment (Ti-C into Ti-O)	Positive shift	Positive shift
Electron filling (existence of $Ti^{\delta+}$)	No change	Negative shift
Comprehensive Result	Positive shift	Negligible shift

Supplementary Table 2 The XPS semi-quantitative data of F contents in OH-terminated Ti_3C_2 and the corresponding electrochemical uranium extraction performance.

OH-terminated Ti_3C_2	F atomic (%)	Extraction efficiency (%)
Poor F^-	8.4	57.5
Moderate F^-	13.7	51.5
Rich F^-	20.1	47.1

Supplementary Table 3 The Optimized adsorption configurations of UO_2F^+ adsorbed on the Ti_3C_2 (OH-terminated), $Ti(OH)PO_4$, and $Ti(OH)PO_4$ with defects.

Substrate	$U-O_{ax}$ (Å)	O-U-O (θ)	$U-O_{eq}$ (Å)	U-F (Å)	Ti-F (Å)	E_{ads} (eV)
U-Ti_3C_2 (OH-terminated)	1.9/1.9	167.4	2.5/2.9	2.2	/	-2.6
U-$Ti(OH)PO_4$	1.8/1.8	100.6	2.2/2.4/2.5	2.6	1.9	-4.5
U-defected $Ti(OH)PO_4$	1.8/2.3	102.6	2.1/2.4	2.4	2.0	-4.9

Supplementary Table 4 The ion concentration of metal species of transparent residual liquid after electrochemical uranium extraction tested by ICP-OES.

Ion species	Concentration (mg L^{-1})	Proportion (%)
K	140939.8	95.908828
Na	6008.6	4.0888222
U	2.2	0.0014971
Mn	0.14	0.0000953
Al	0.023	0.0000157
Fe	1.00	0.0006805
Ca	0.09	0.0000612

Supplementary References

1. Xue, Q., Zhang, H., Zhu, M., Pei, Z., Li, H., Wang, Z., Huang, Y., Huang, Y., Deng, Q., Zhou, J., Du, S., Huang, Q. & Zhi, C. Photoluminescent Ti_3C_2 MXene quantum dots for multicolor cellular imaging. *Adv. Mater.* **29**, 1604847 (2017).
2. Zhou, K., Gong, K., Gao, F. & Yin, L. Facile strategy to synthesize MXene@LDH nanohybrids for boosting the flame retardancy and smoke suppression properties of epoxy. *Compos. Part A Appl. Sci. Manuf.* **157**, 106912 (2022).
