# Supplementary material for: Ion pair sites for efficient electrochemical extraction of uranium in real nuclear wastewater
Source: Nat Commun. 2024 May 16;15:4149. doi: 10.1038/s41467-024-48564-y (PMC11099191; doi:10.1038/s41467-024-48564-y)
Supplement: Supplementary file 3 — Description of Additional Supplementary Files [file 41467_2024_48564_MOESM3_ESM.pdf]

Description of Additional Supplementary Files

File Name: Supplementary Data 1
Description: The atomic coordinates of the optimized structures in related DFT calculations.
