# Supplementary figures and images for: Ion pair sites for efficient electrochemical extraction of uranium in real nuclear wastewater
Source: Nat Commun. 2024 May 16;15:4149. doi: 10.1038/s41467-024-48564-y (PMC11099191; doi:10.1038/s41467-024-48564-y)

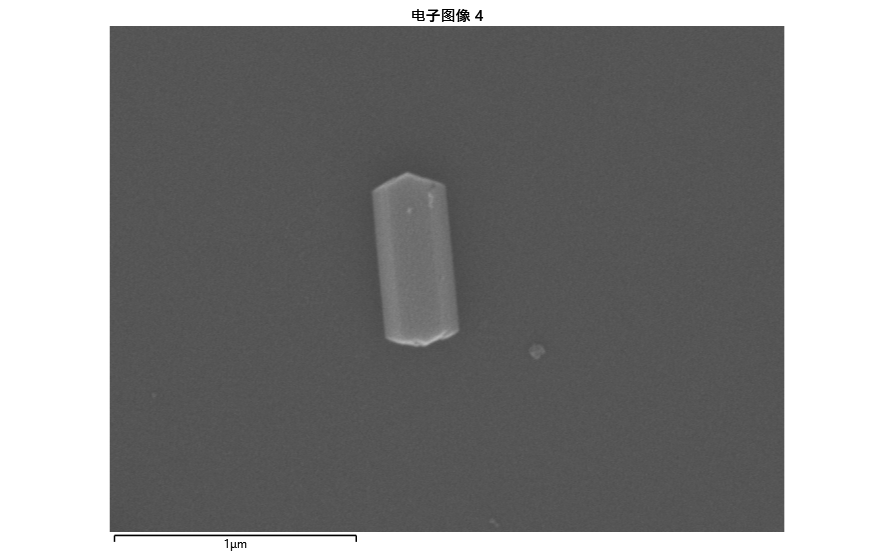


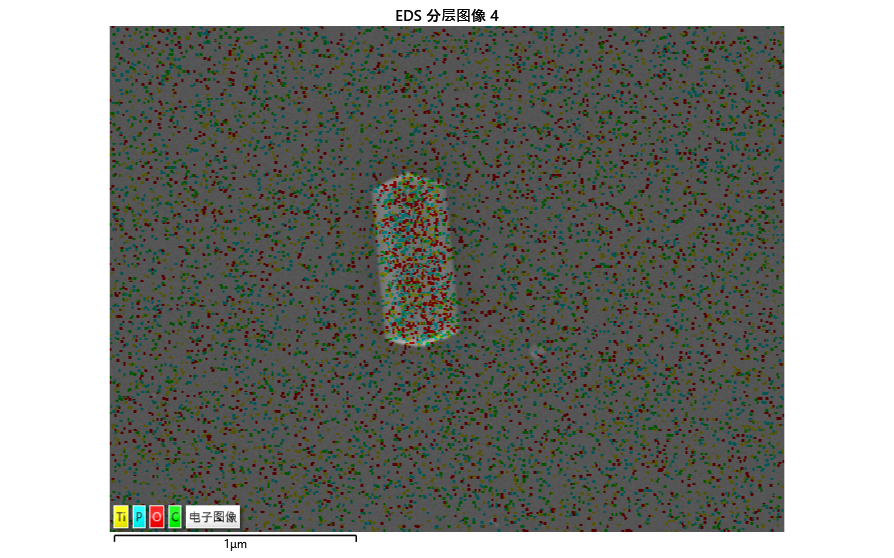


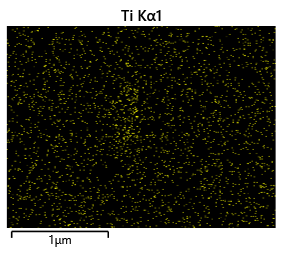


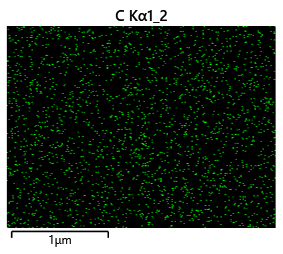

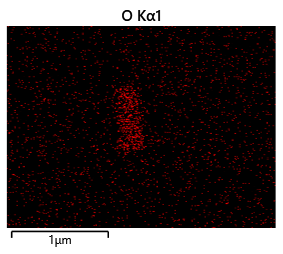

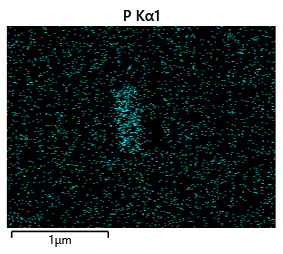

Supplement: Supplementary file 5 — Source Data [file 41467_2024_48564_MOESM5_ESM.zip › Source Data/Fig. 1/Fig. 1d.docx]

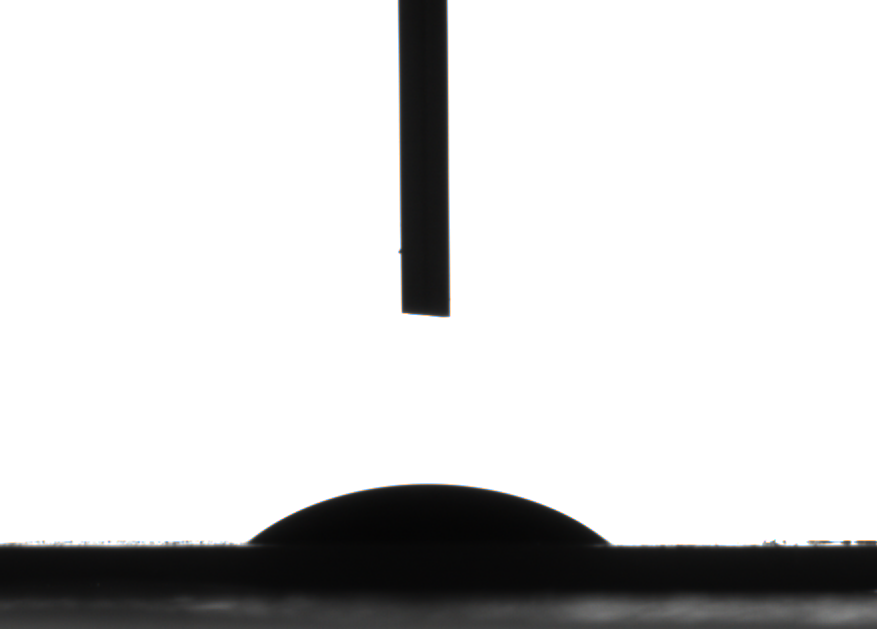

Supplement: Supplementary file 5 — Source Data [file 41467_2024_48564_MOESM5_ESM.zip › Source Data/Fig. 1/Fig. 1g/OH-terminated Ti3C2.bmp]

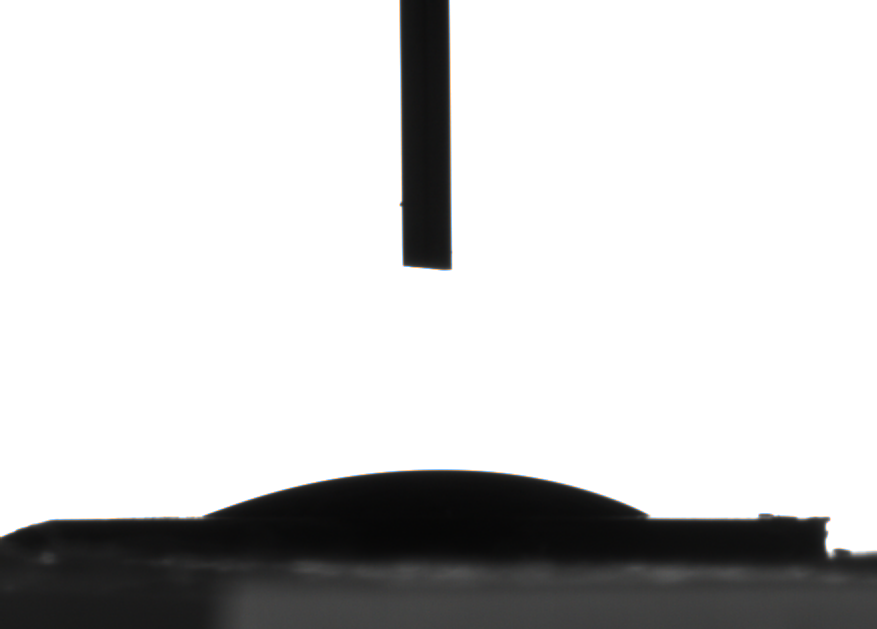

Supplement: Supplementary file 5 — Source Data [file 41467_2024_48564_MOESM5_ESM.zip › Source Data/Fig. 1/Fig. 1g/Ti(OH)PO4.bmp]

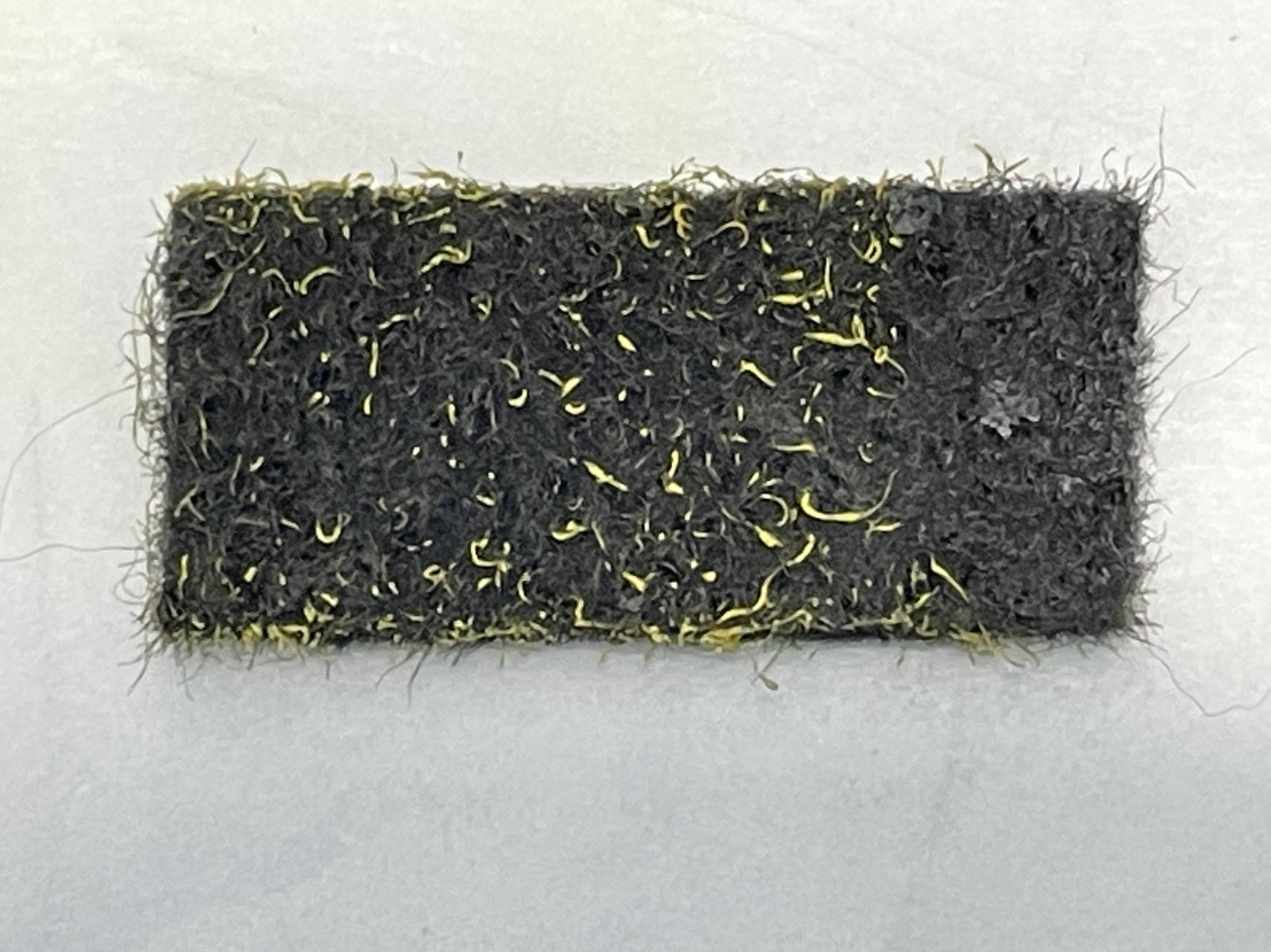

Supplement: Supplementary file 5 — Source Data [file 41467_2024_48564_MOESM5_ESM.zip › Source Data/Fig. 3/Fig. 3b (Inset).jpg]

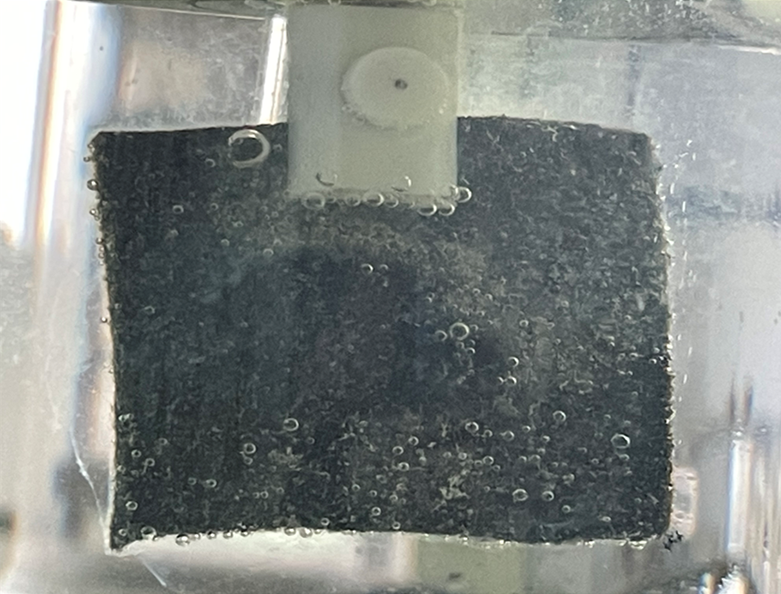

Supplement: Supplementary file 5 — Source Data [file 41467_2024_48564_MOESM5_ESM.zip › Source Data/Fig. 4/Fig. 4a/Fig. 4a 1h.tif]

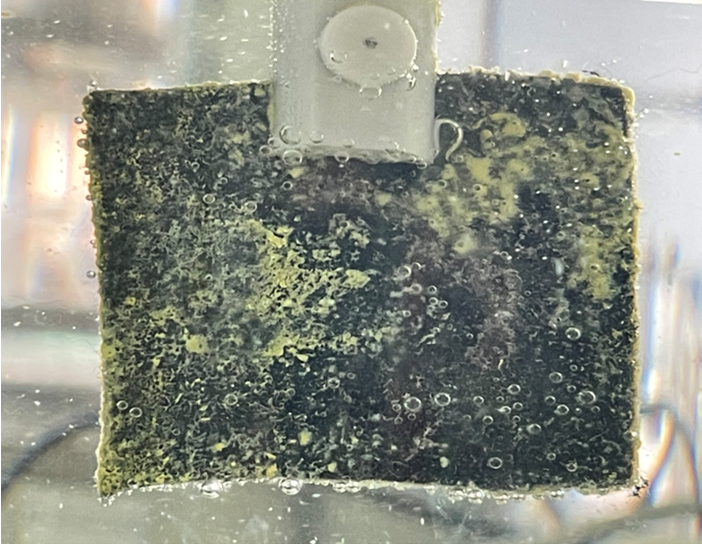

Supplement: Supplementary file 5 — Source Data [file 41467_2024_48564_MOESM5_ESM.zip › Source Data/Fig. 4/Fig. 4a/Fig. 4a 3h.tif]

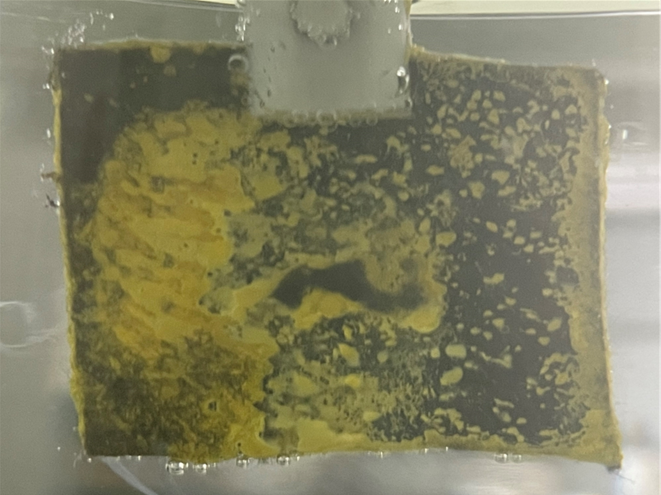

Supplement: Supplementary file 5 — Source Data [file 41467_2024_48564_MOESM5_ESM.zip › Source Data/Fig. 4/Fig. 4a/Fig. 4a 5h.tif]

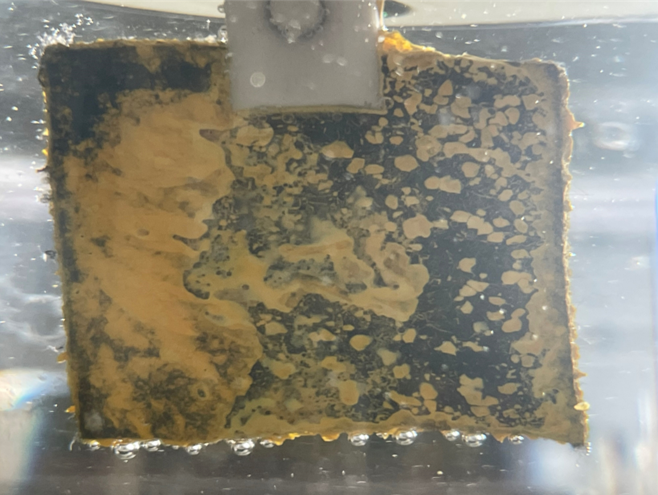

Supplement: Supplementary file 5 — Source Data [file 41467_2024_48564_MOESM5_ESM.zip › Source Data/Fig. 4/Fig. 4a/Fig. 4a 7h.tif]

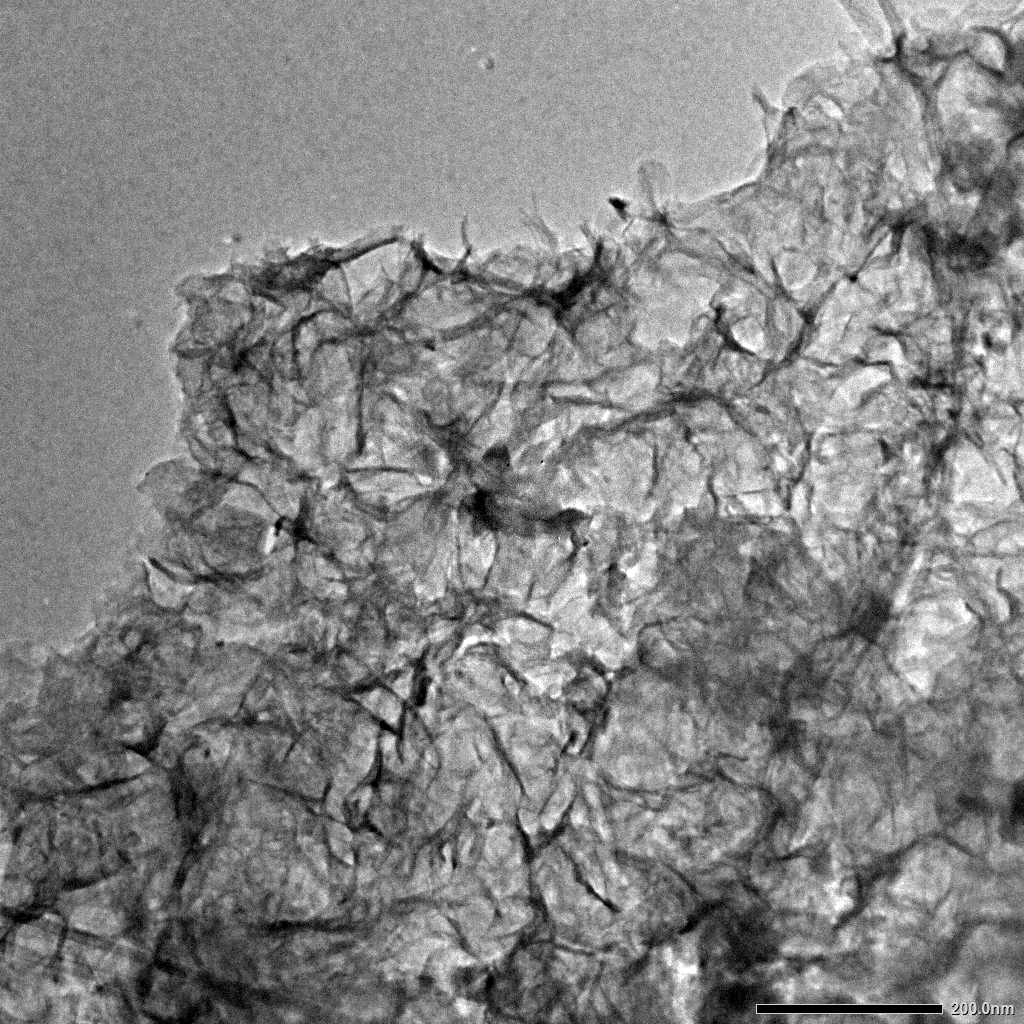

Supplement: Supplementary file 5 — Source Data [file 41467_2024_48564_MOESM5_ESM.zip › Source Data/Fig. 4/Fig. 4d.bmp]

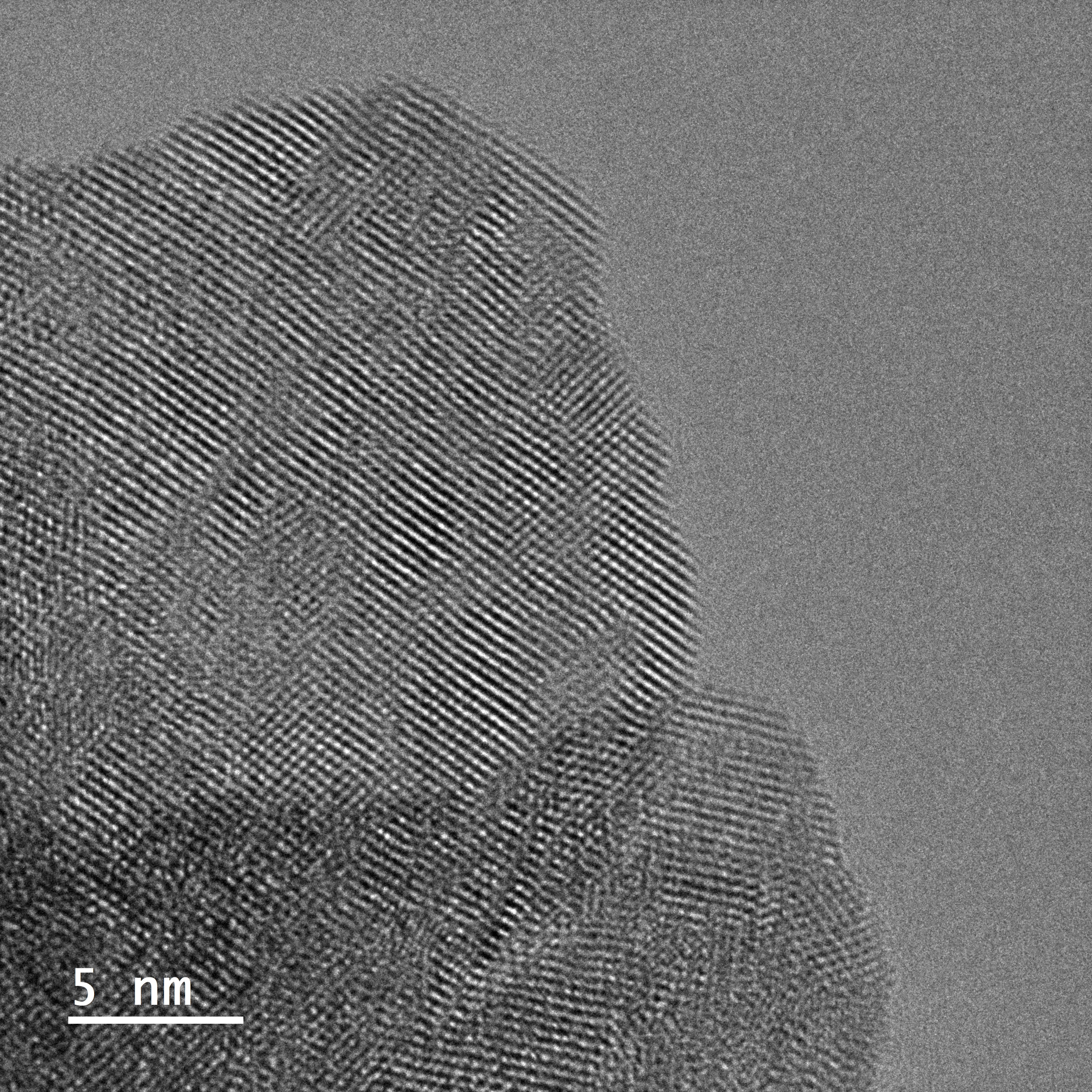

Supplement: Supplementary file 5 — Source Data [file 41467_2024_48564_MOESM5_ESM.zip › Source Data/Fig. 4/Fig. 4e.jpg]

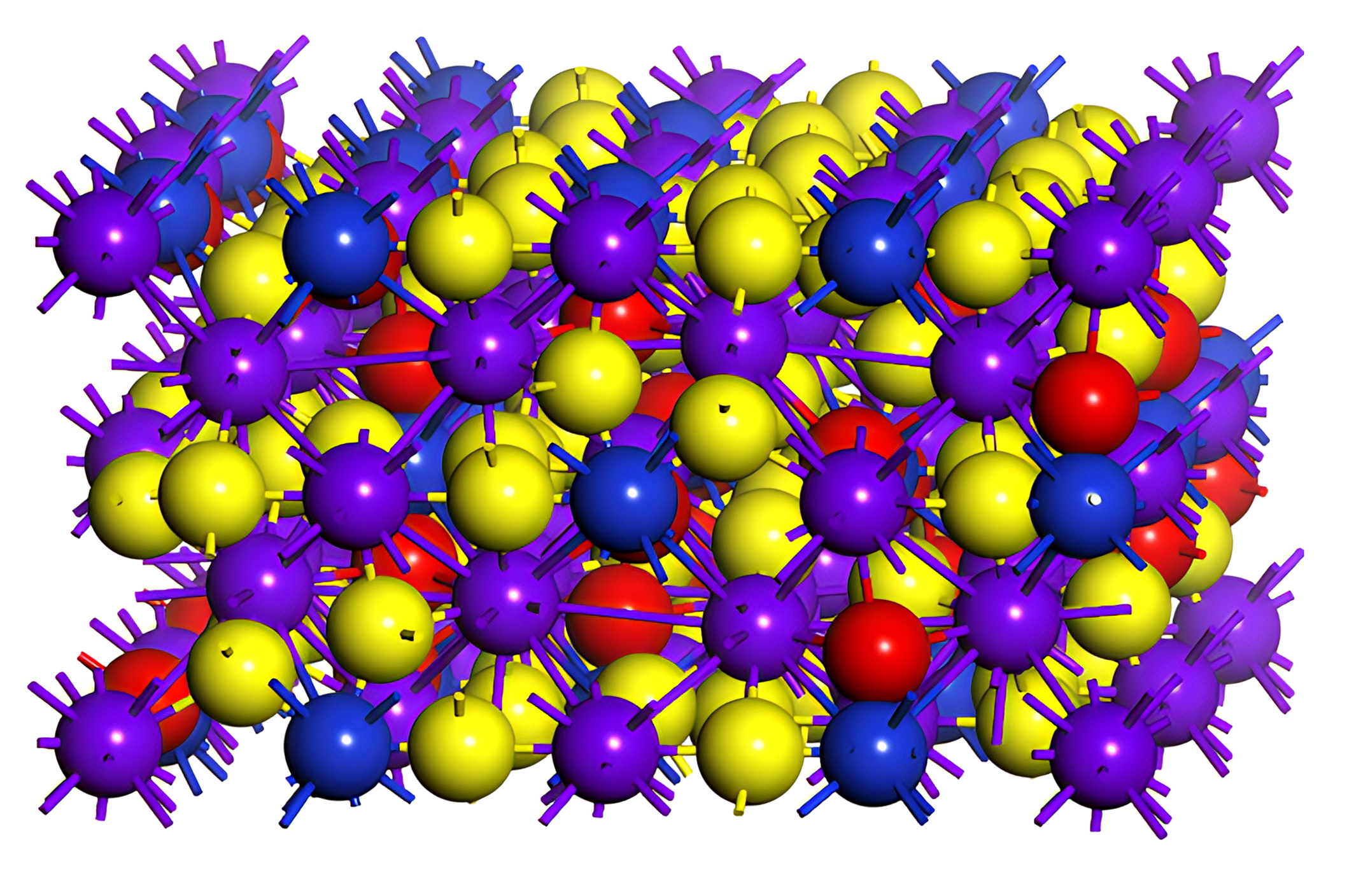

Supplement: Supplementary file 5 — Source Data [file 41467_2024_48564_MOESM5_ESM.zip › Source Data/Fig. 5/Fig. 5a/Model of K3UO2F5.tif]

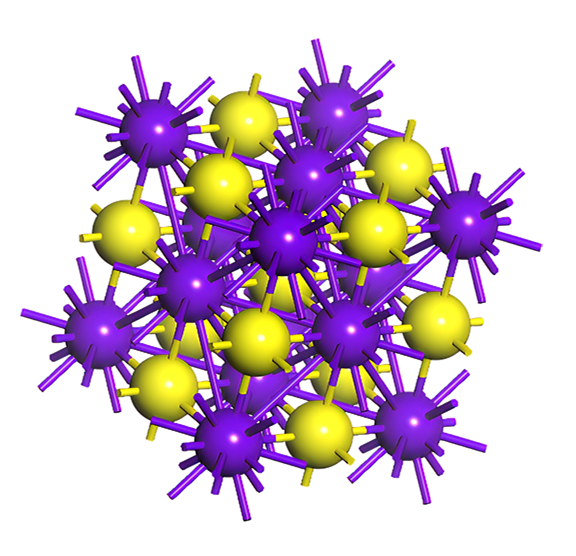

Supplement: Supplementary file 5 — Source Data [file 41467_2024_48564_MOESM5_ESM.zip › Source Data/Fig. 5/Fig. 5a/Model of KF.tif]

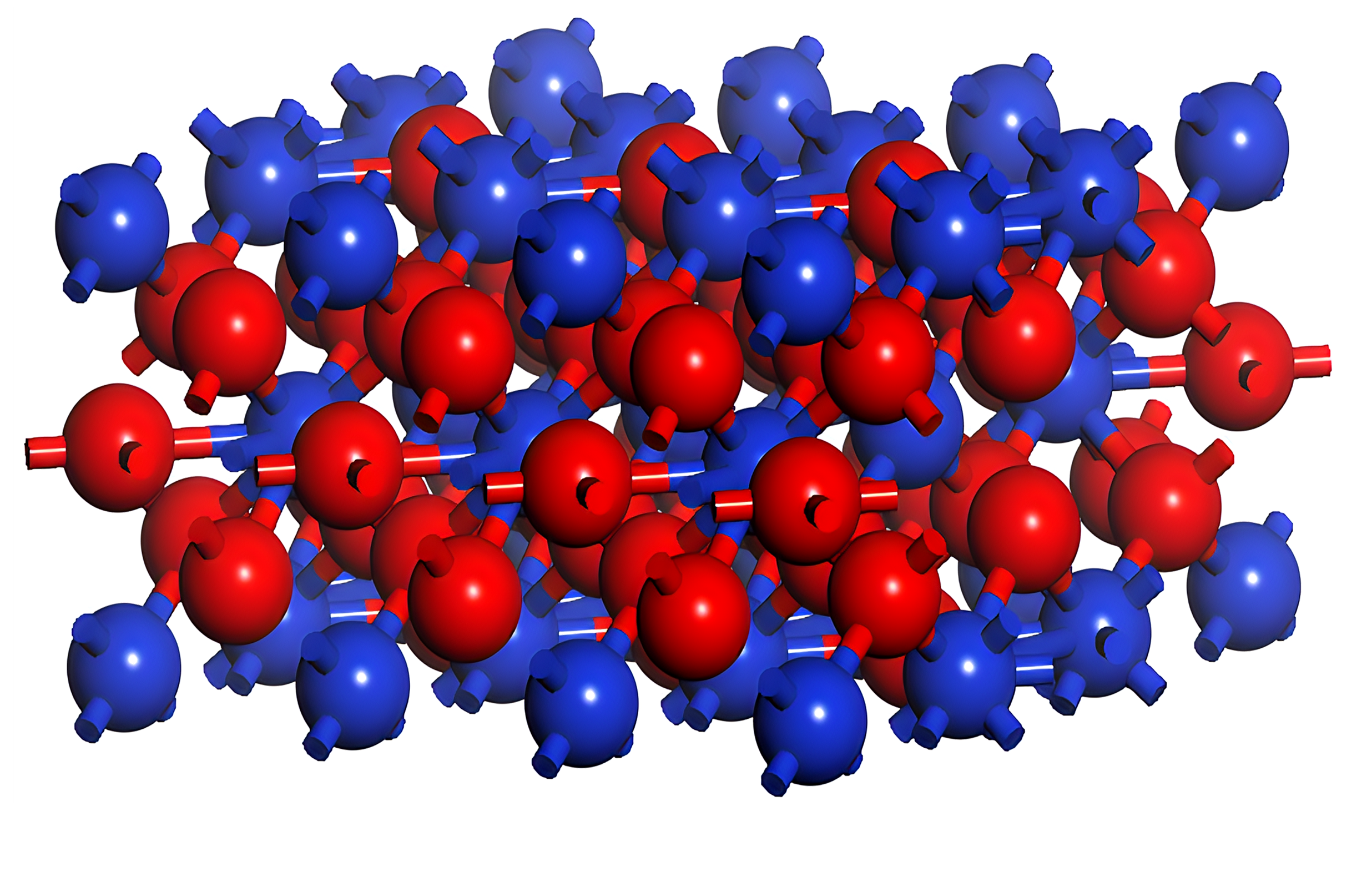

Supplement: Supplementary file 5 — Source Data [file 41467_2024_48564_MOESM5_ESM.zip › Source Data/Fig. 5/Fig. 5a/Model of U3O7.tif]

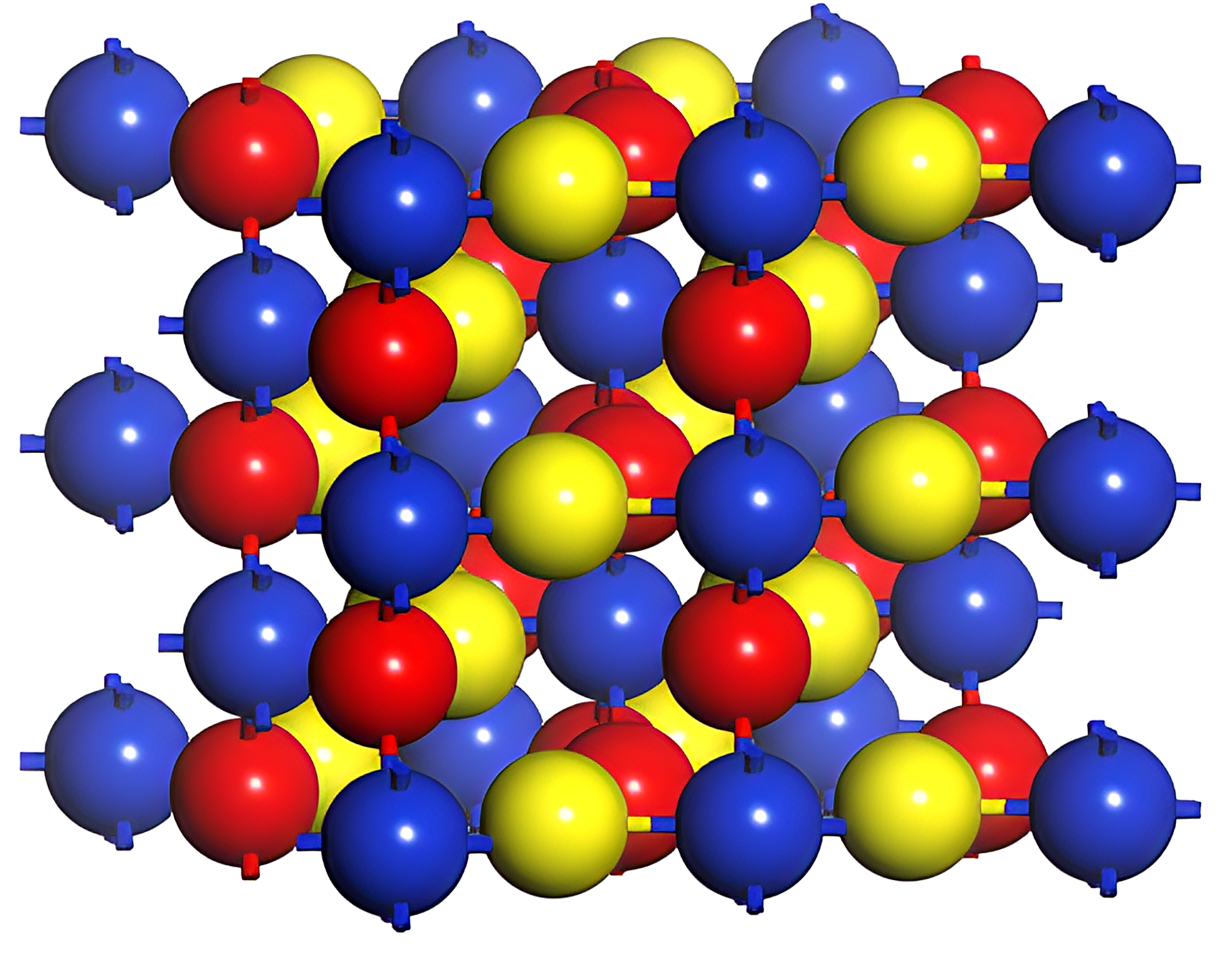

Supplement: Supplementary file 5 — Source Data [file 41467_2024_48564_MOESM5_ESM.zip › Source Data/Fig. 5/Fig. 5a/Model of UO2Fx.tif]

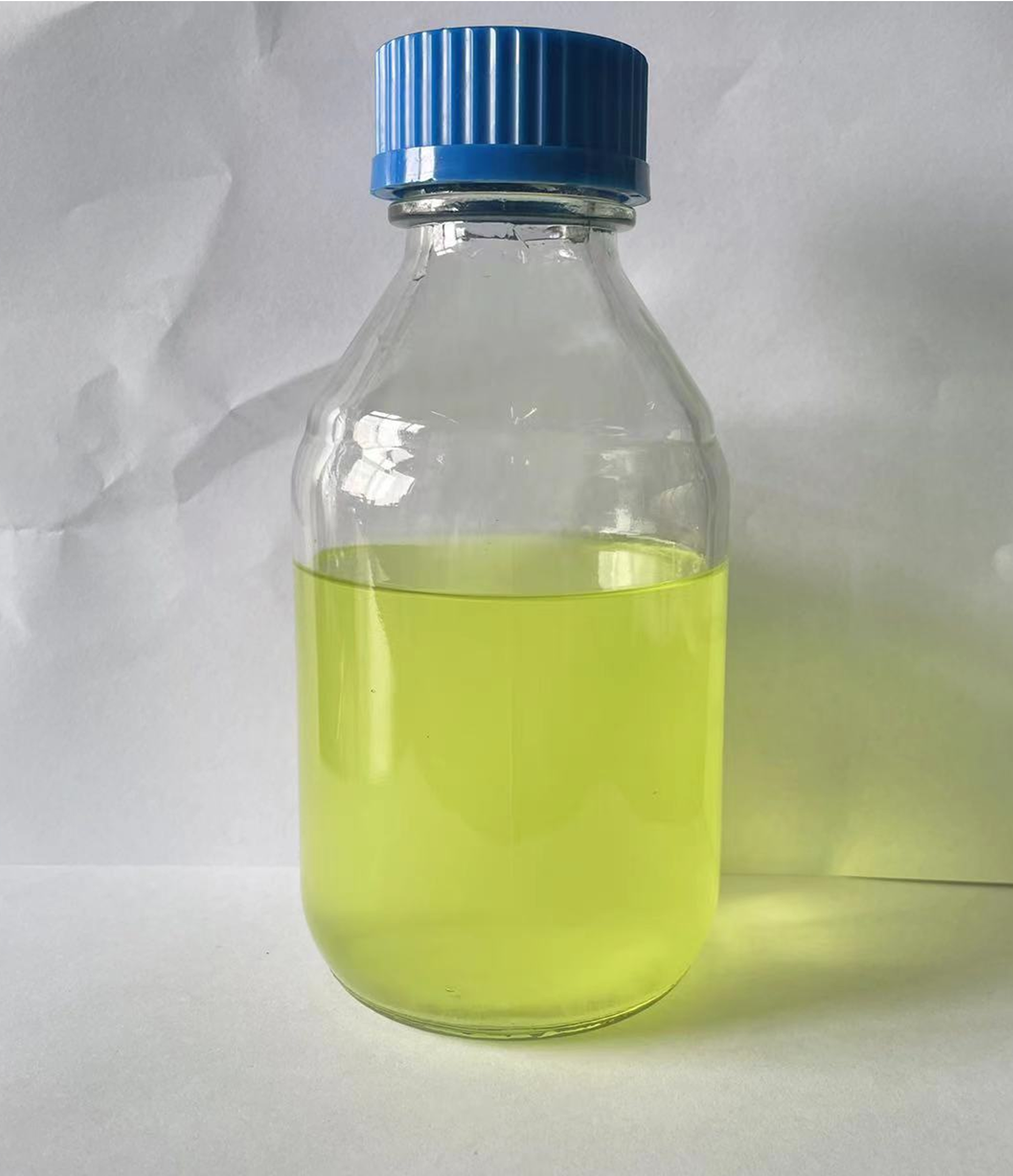

Supplement: Supplementary file 5 — Source Data [file 41467_2024_48564_MOESM5_ESM.zip › Source Data/Fig. 6/Fig. 6b/Fig. 6b real nuclear wastewater.tif]

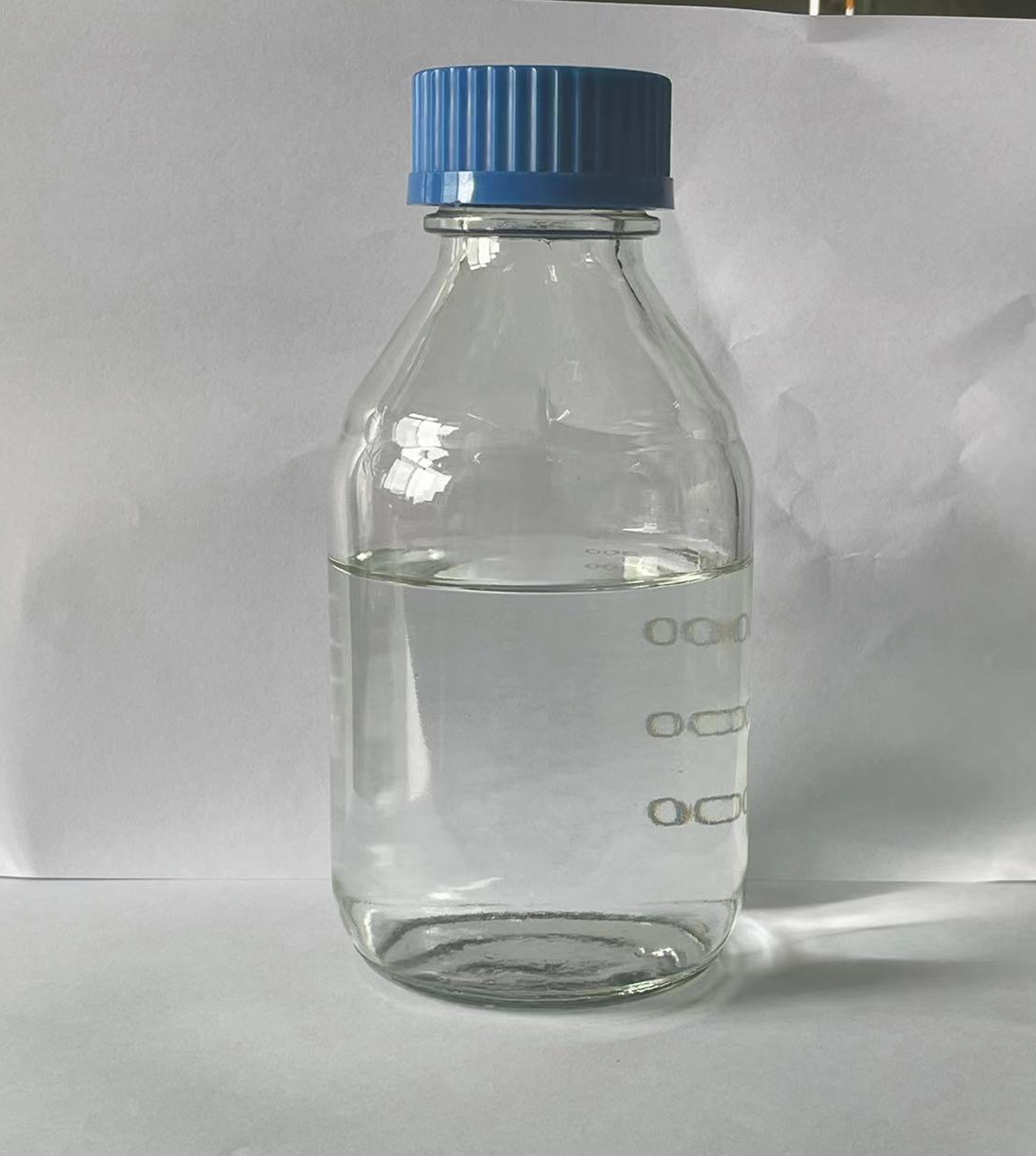

Supplement: Supplementary file 5 — Source Data [file 41467_2024_48564_MOESM5_ESM.zip › Source Data/Fig. 6/Fig. 6b/Fig. 6b residual liquid .tif]

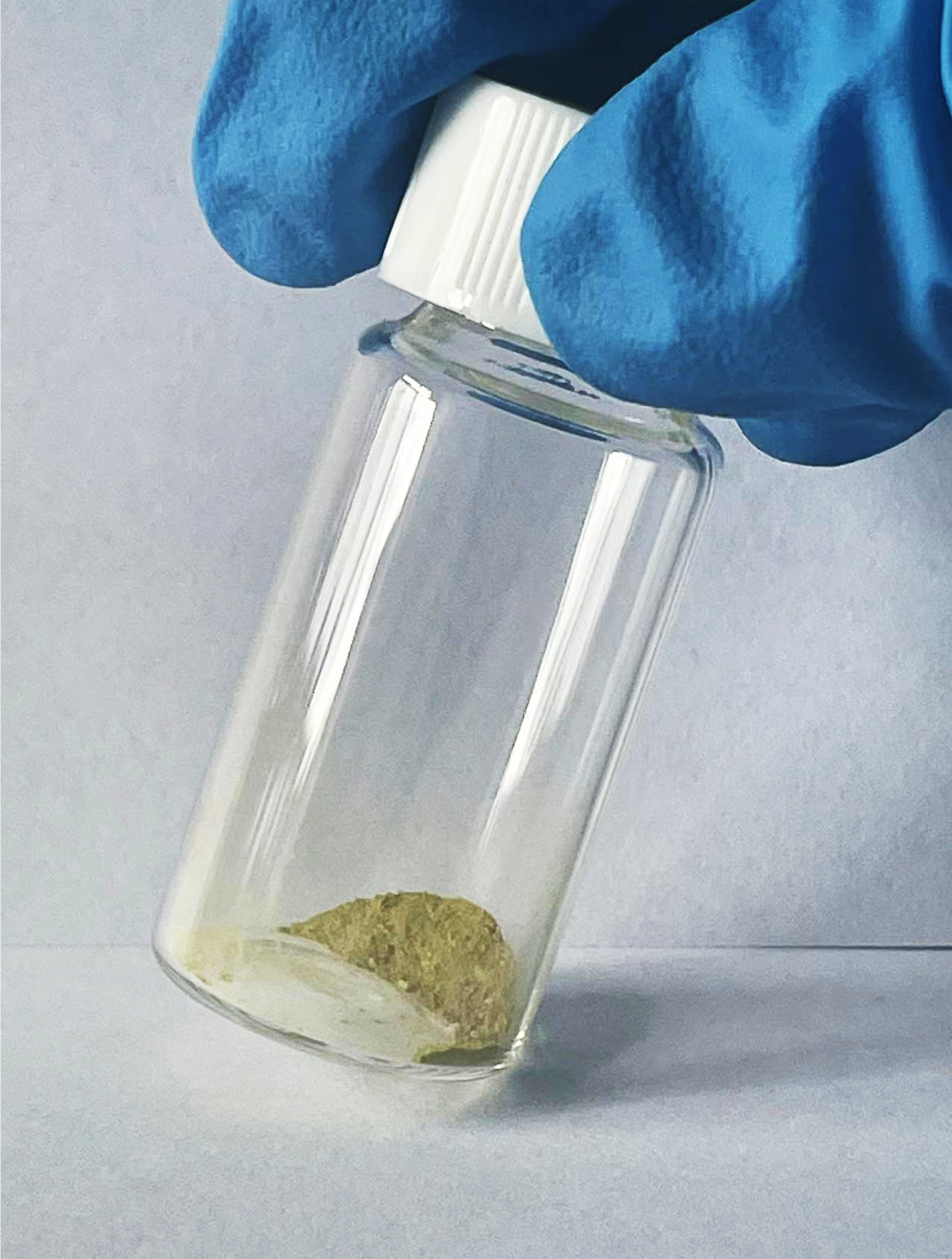

Supplement: Supplementary file 5 — Source Data [file 41467_2024_48564_MOESM5_ESM.zip › Source Data/Fig. 6/Fig. 6c/Fig. 6c collected powder.tif]

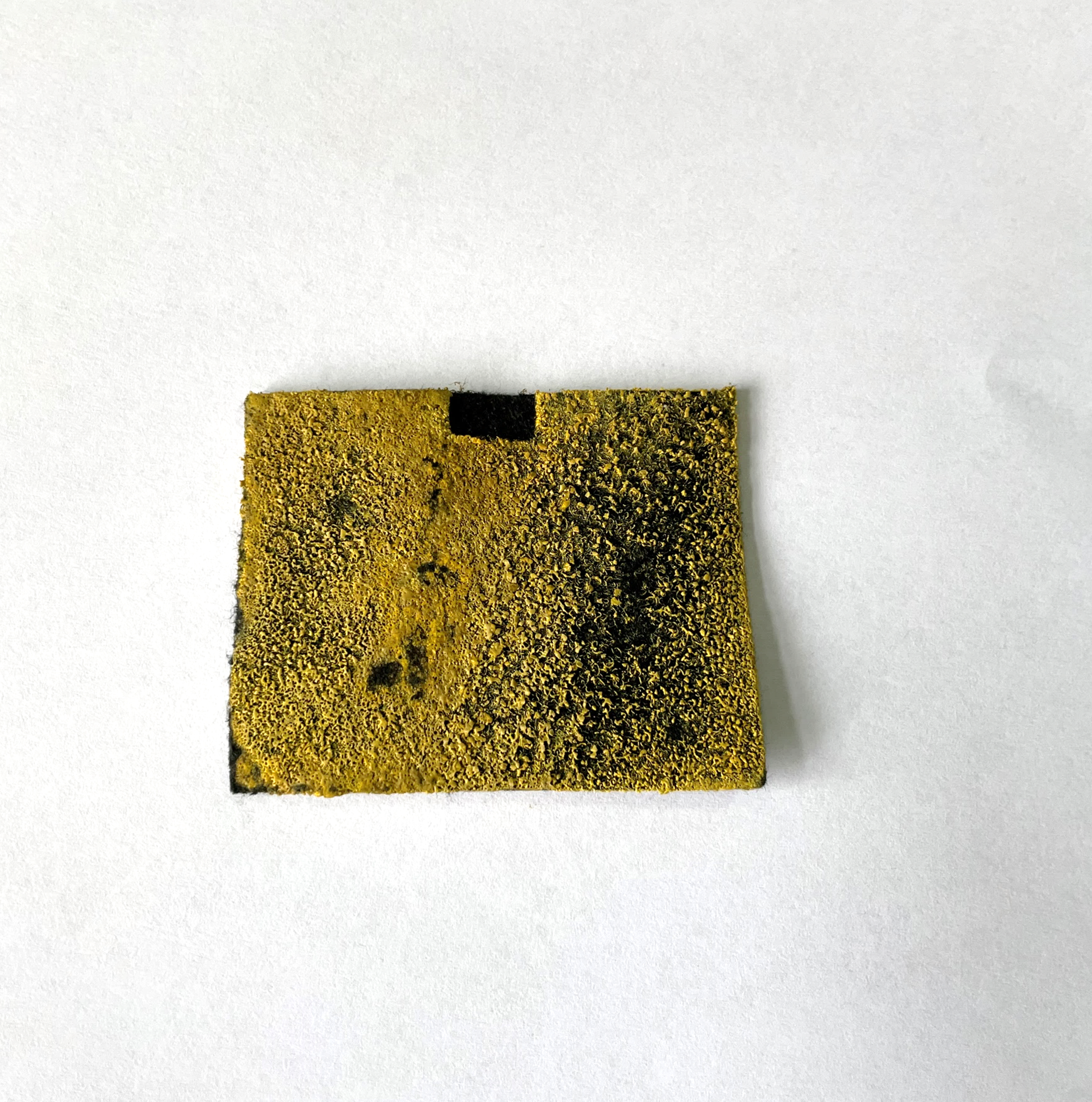

Supplement: Supplementary file 5 — Source Data [file 41467_2024_48564_MOESM5_ESM.zip › Source Data/Fig. 6/Fig. 6c/Fig. 6c electrode after electrolysis .tif]

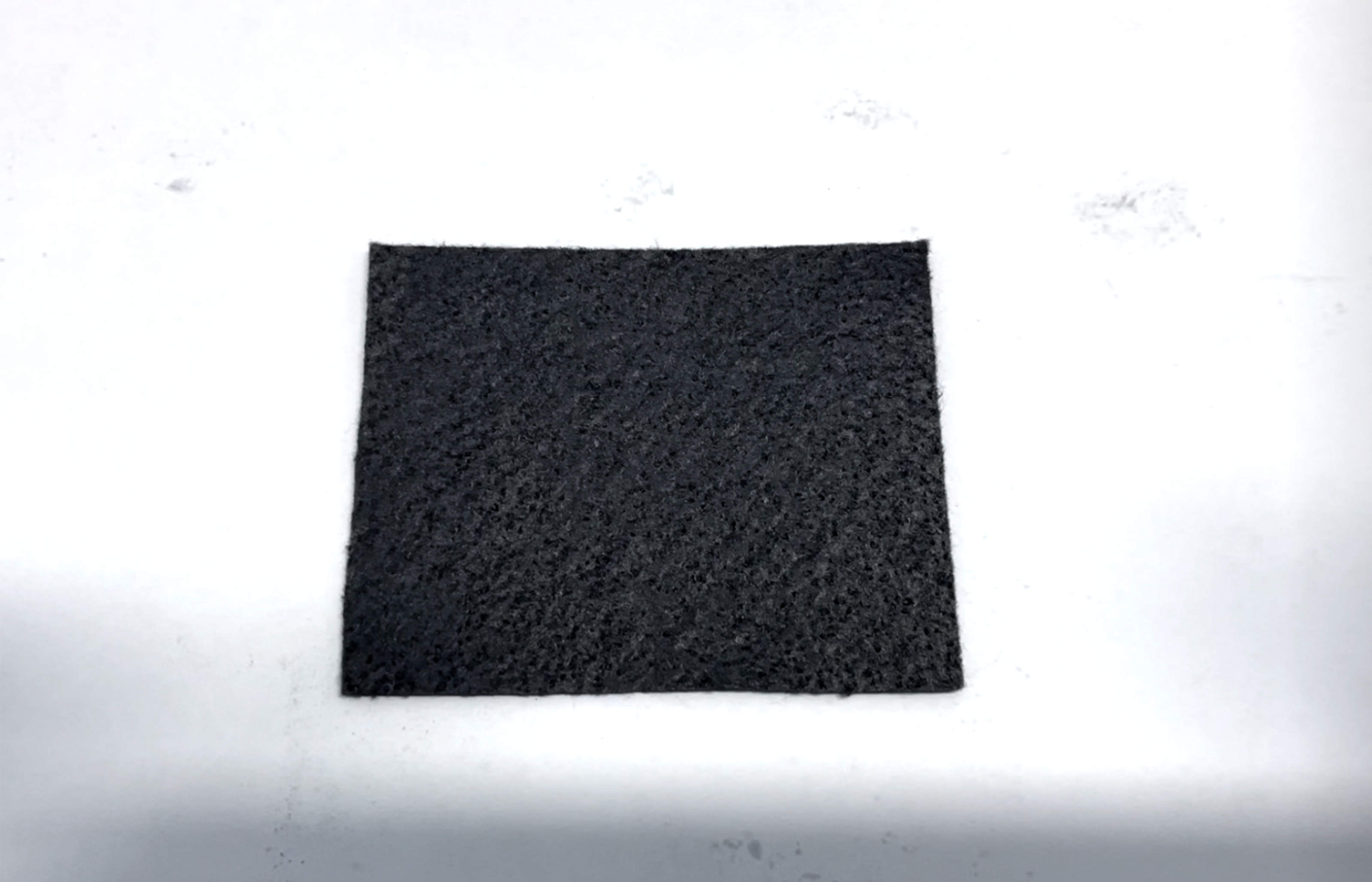

Supplement: Supplementary file 5 — Source Data [file 41467_2024_48564_MOESM5_ESM.zip › Source Data/Fig. 6/Fig. 6c/Fig. 6c electrode.tif]

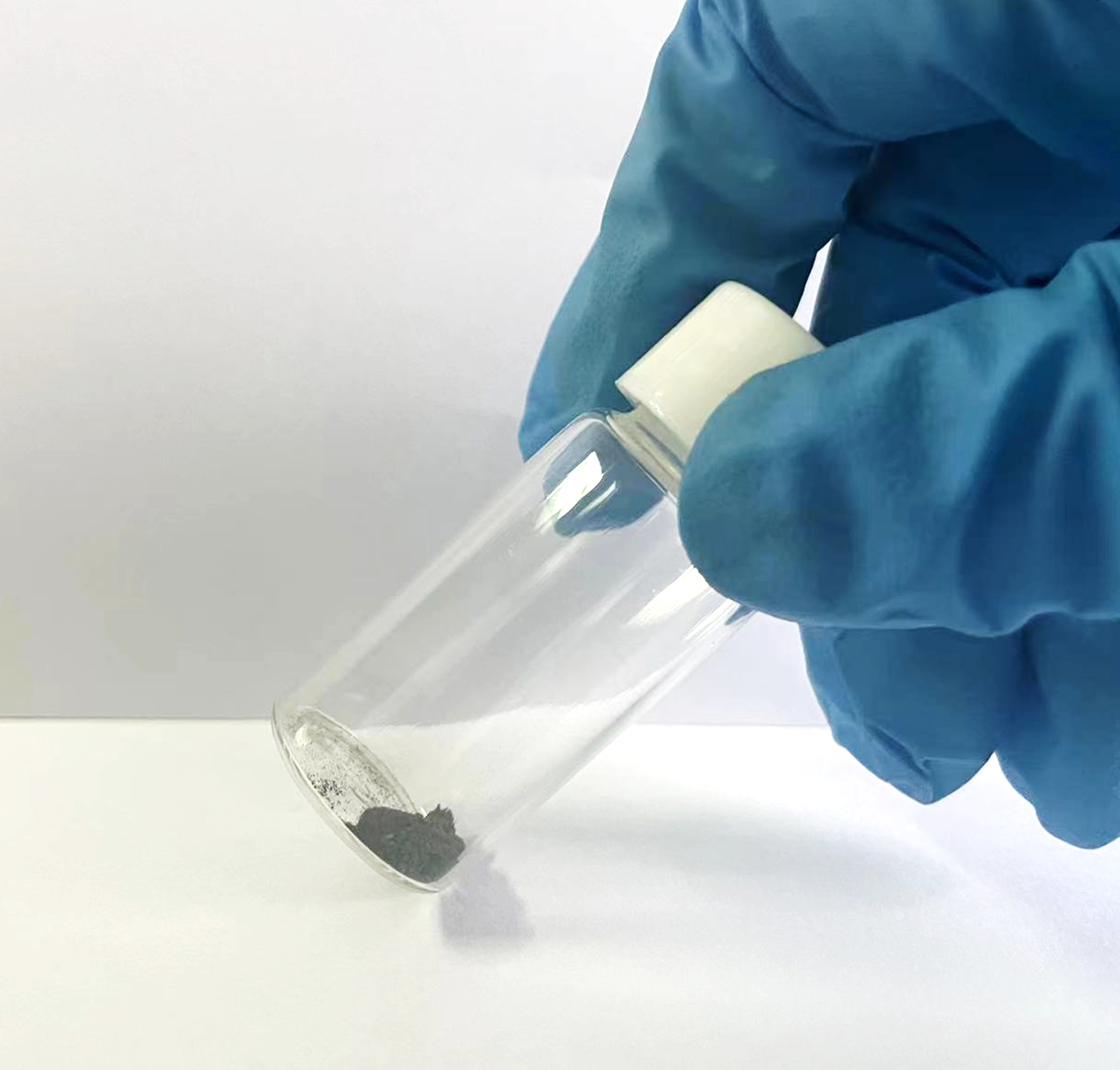

Supplement: Supplementary file 5 — Source Data [file 41467_2024_48564_MOESM5_ESM.zip › Source Data/Fig. 6/Fig. 6e/Fig. 6e U3O8.tif]

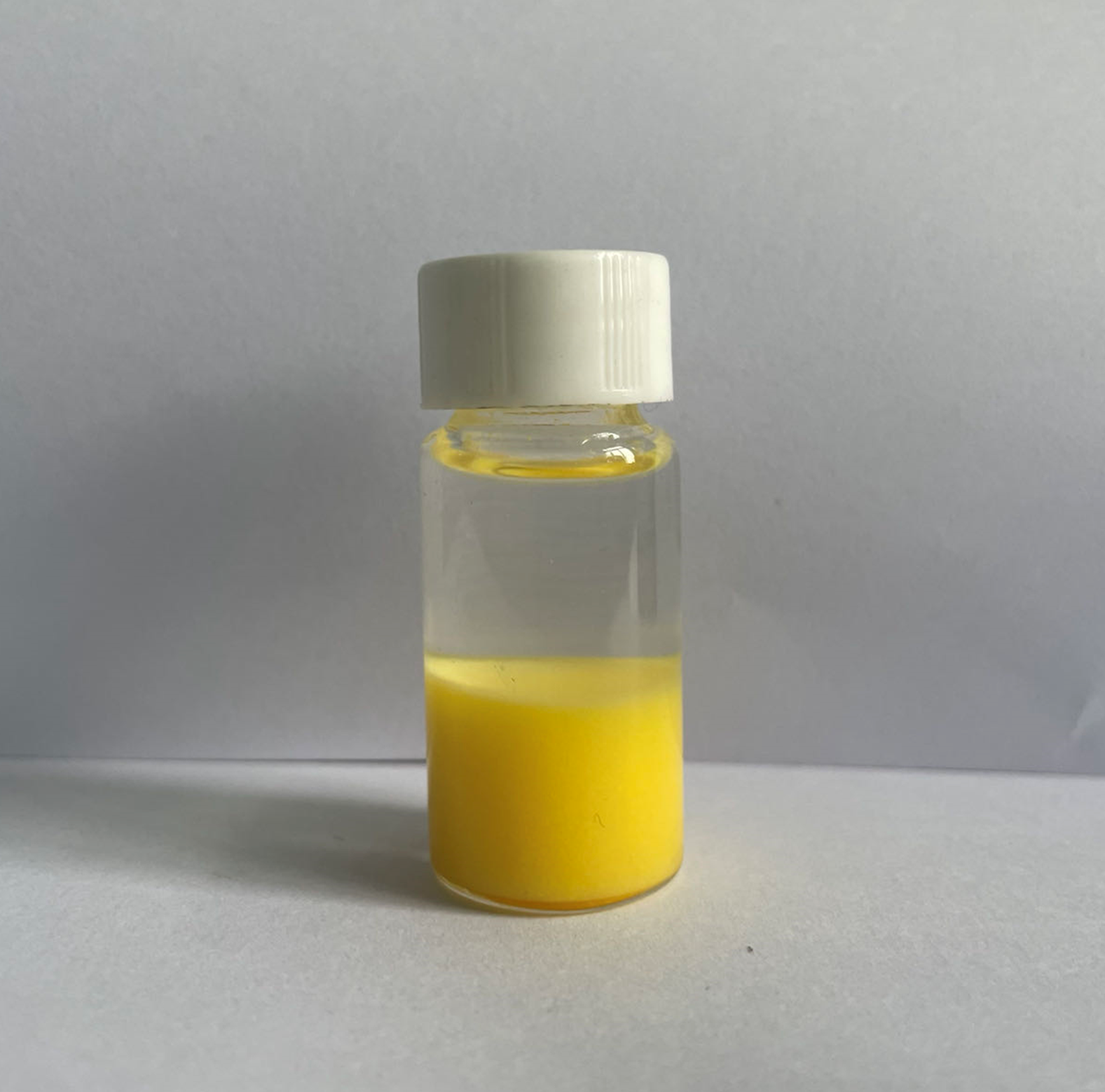

Supplement: Supplementary file 5 — Source Data [file 41467_2024_48564_MOESM5_ESM.zip › Source Data/Fig. 6/Fig. 6e/Fig. 6e precipitate.tif]

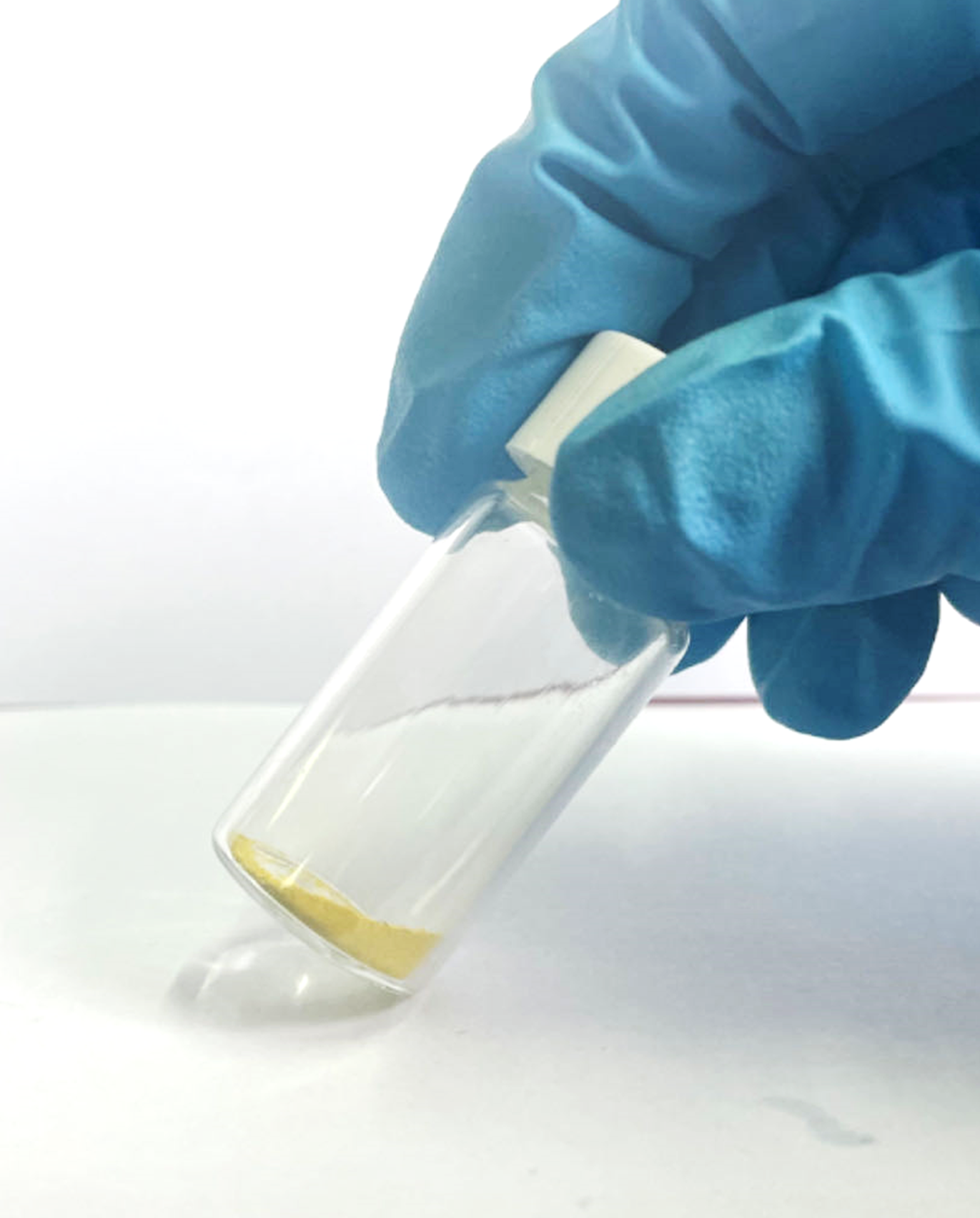

Supplement: Supplementary file 5 — Source Data [file 41467_2024_48564_MOESM5_ESM.zip › Source Data/Fig. 6/Fig. 6e/Fig. 6e yellow powder.tif]

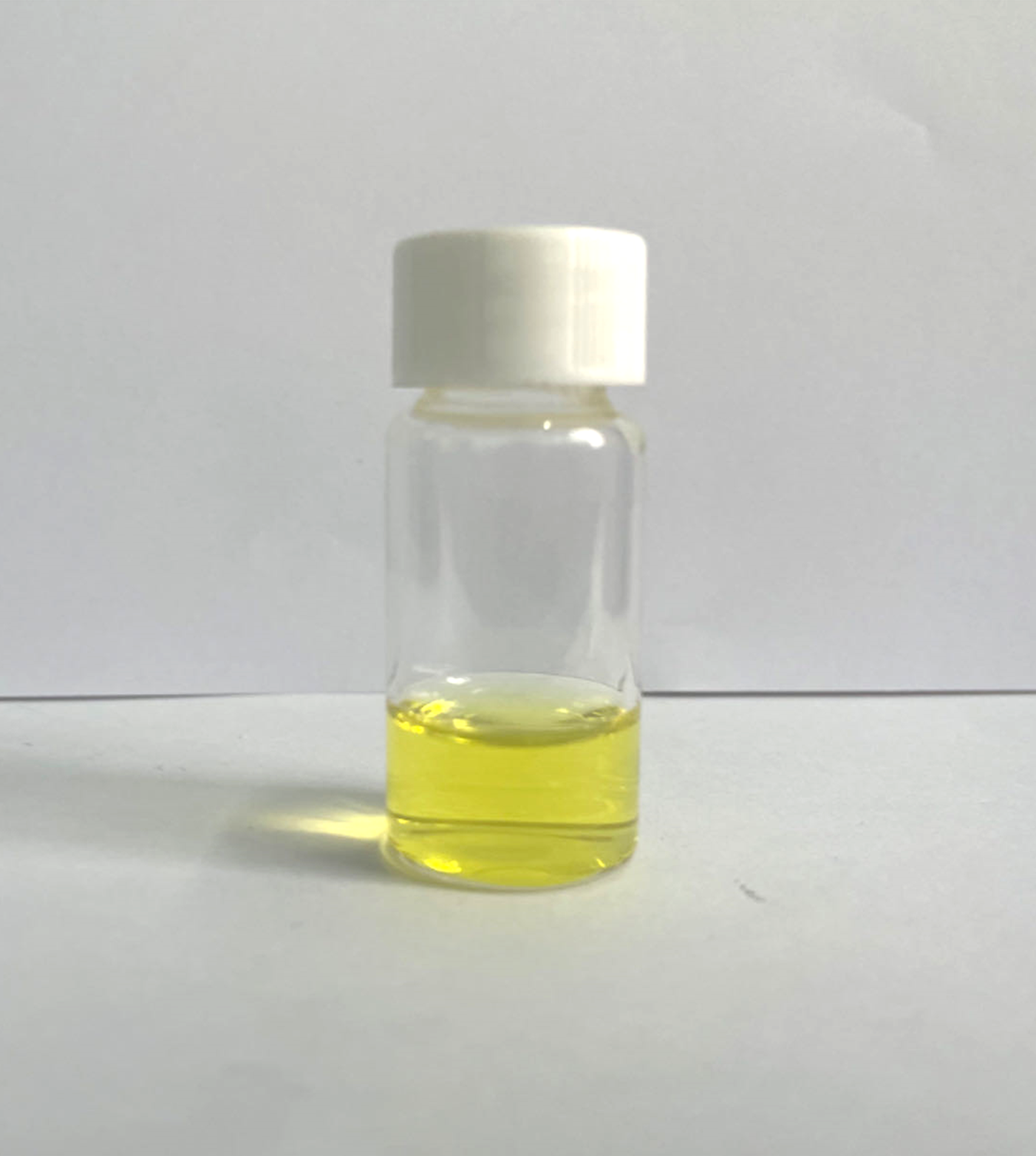

Supplement: Supplementary file 5 — Source Data [file 41467_2024_48564_MOESM5_ESM.zip › Source Data/Fig. 6/Fig. 6e/Fig. 6e yellow solution.tif]

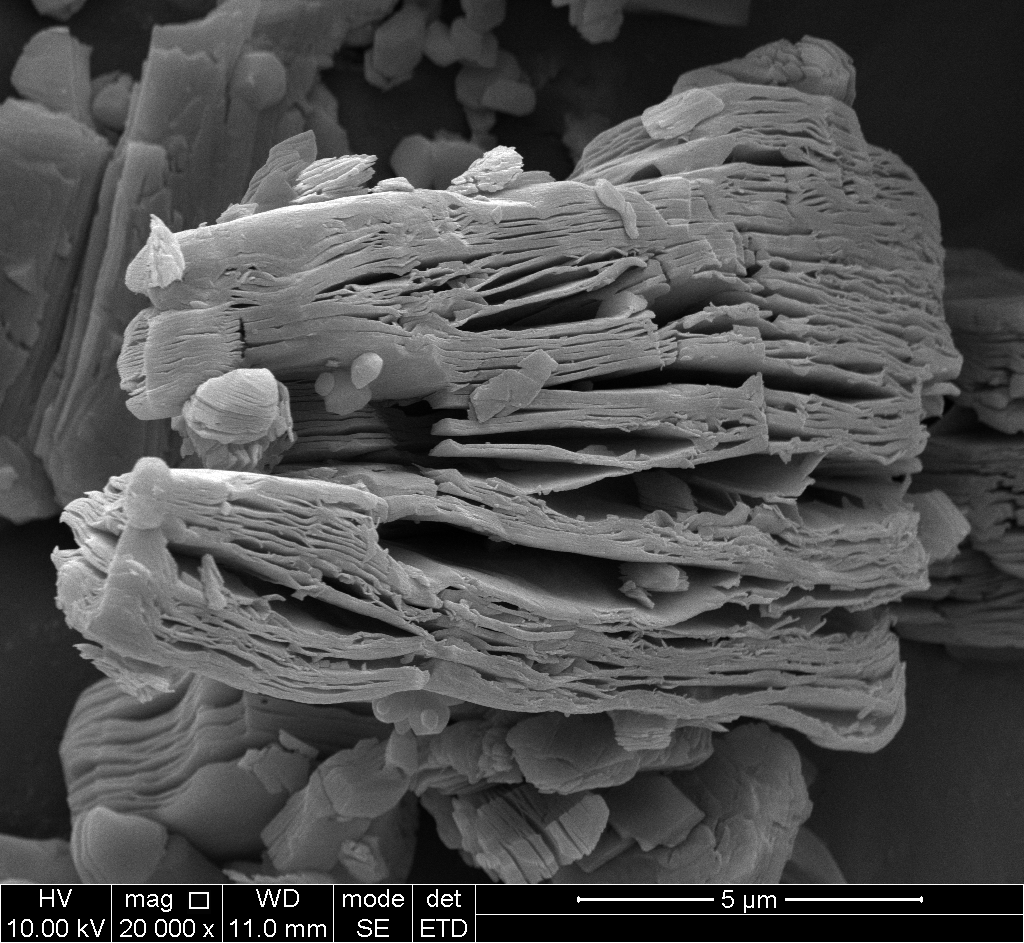

Supplement: Supplementary file 5 — Source Data [file 41467_2024_48564_MOESM5_ESM.zip › Source Data/Supplementary Fig. 1/Accordion-like Ti3C2.tif]

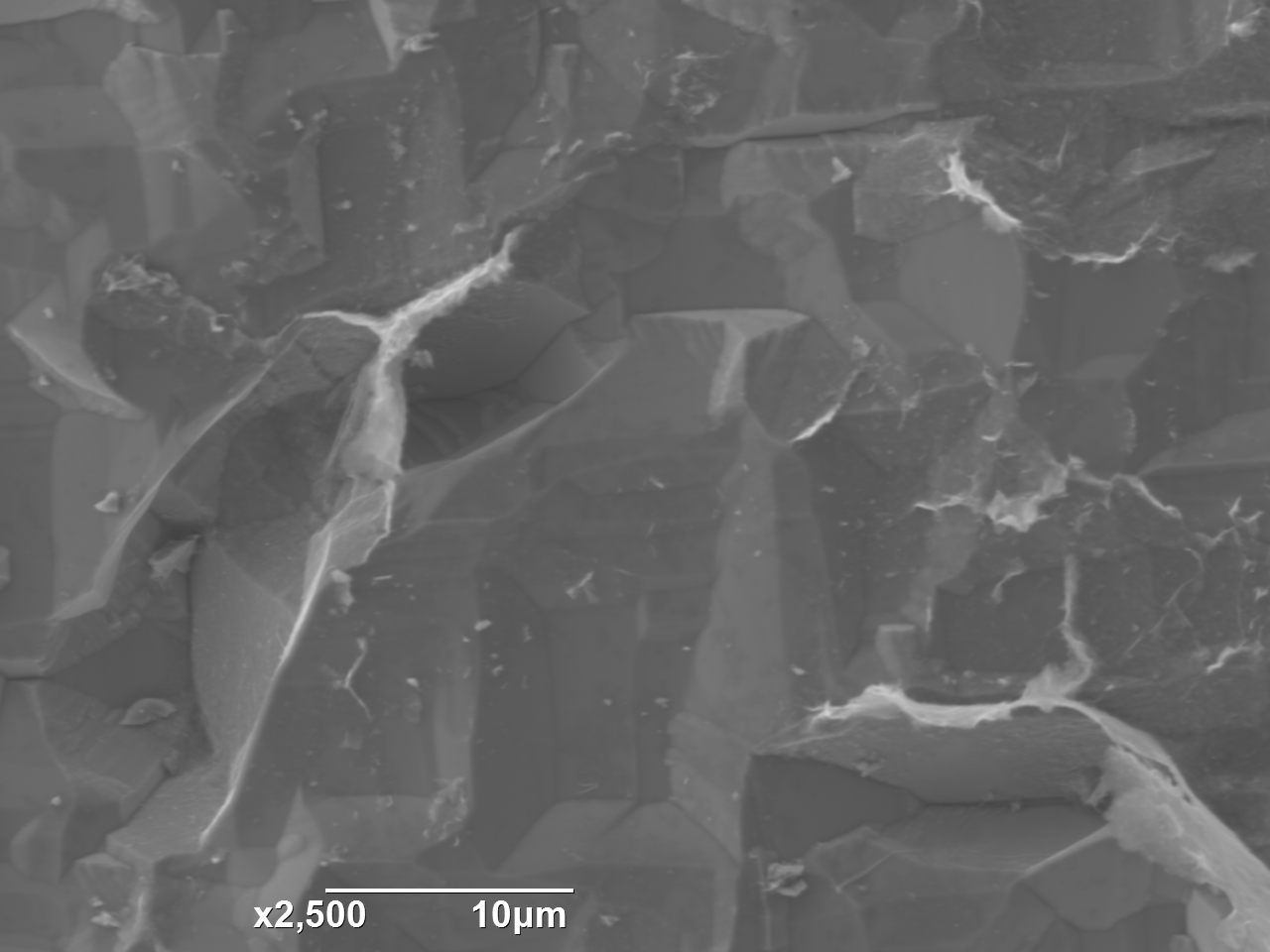

Supplement: Supplementary file 5 — Source Data [file 41467_2024_48564_MOESM5_ESM.zip › Source Data/Supplementary Fig. 1/Exfoliated Ti3C2.bmp]

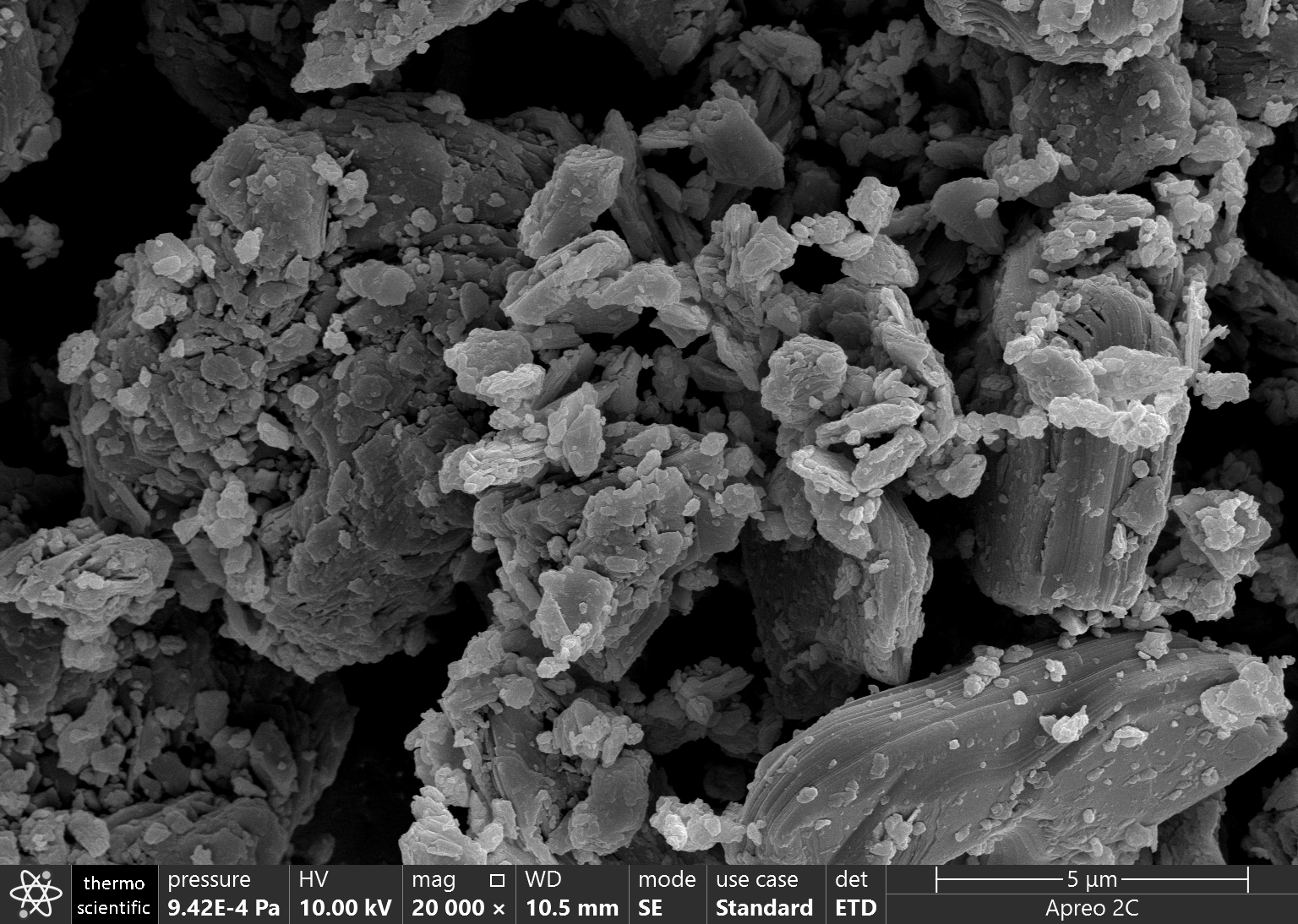

Supplement: Supplementary file 5 — Source Data [file 41467_2024_48564_MOESM5_ESM.zip › Source Data/Supplementary Fig. 1/Ti3AlC2 bulk.tif]

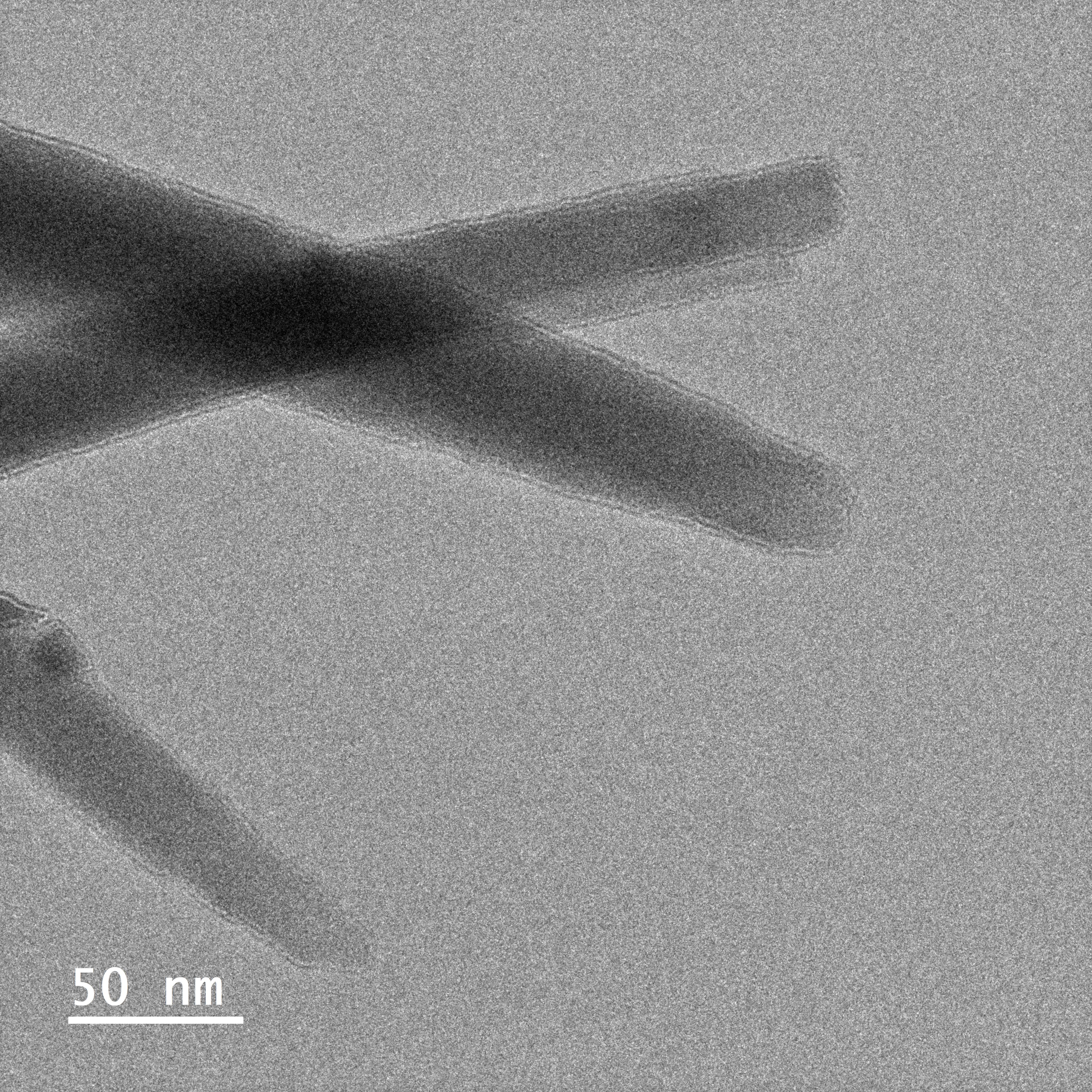

Supplement: Supplementary file 5 — Source Data [file 41467_2024_48564_MOESM5_ESM.zip › Source Data/Supplementary Fig. 16/Supplementary Fig. 16a.jpg]

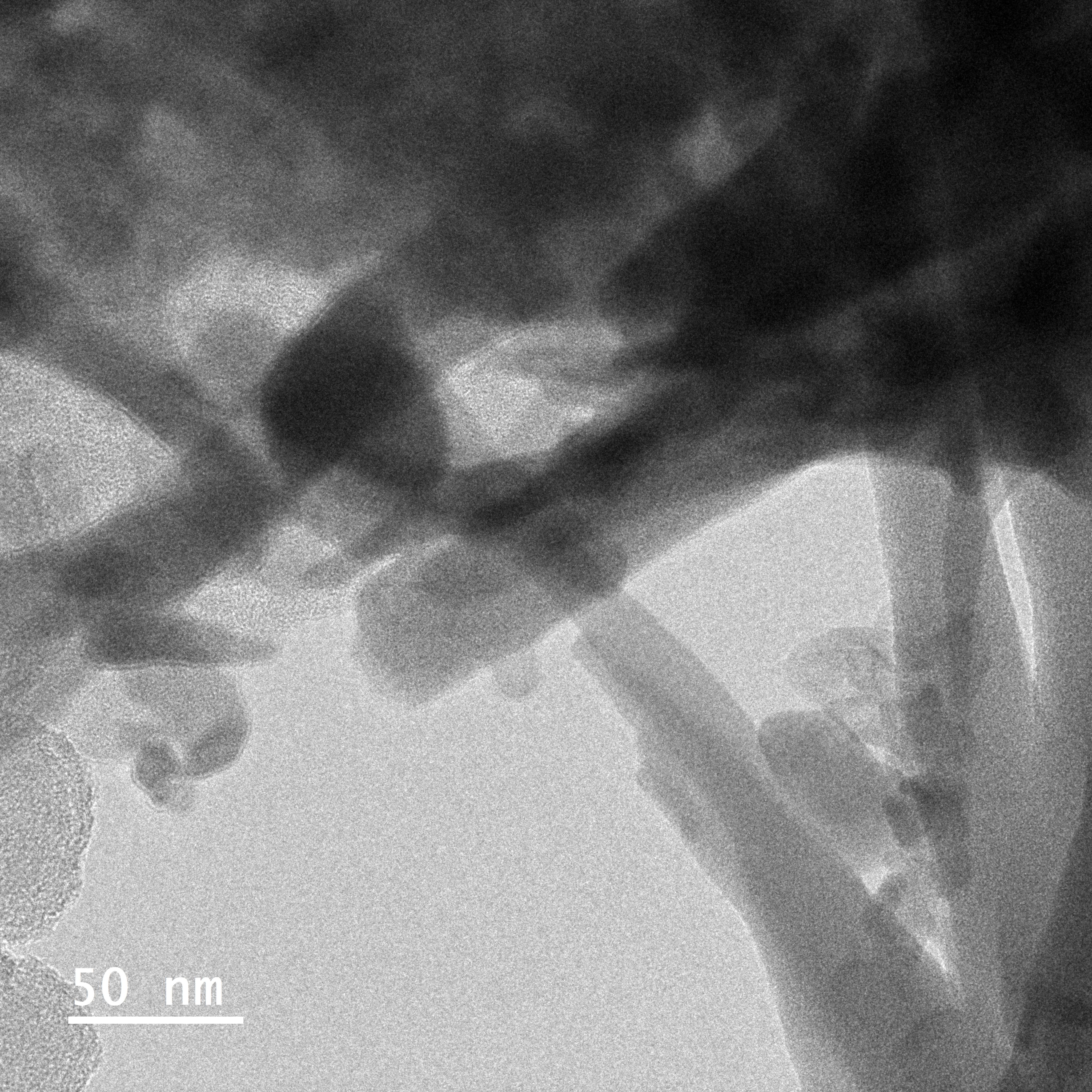

Supplement: Supplementary file 5 — Source Data [file 41467_2024_48564_MOESM5_ESM.zip › Source Data/Supplementary Fig. 16/Supplementary Fig. 16b.jpg]

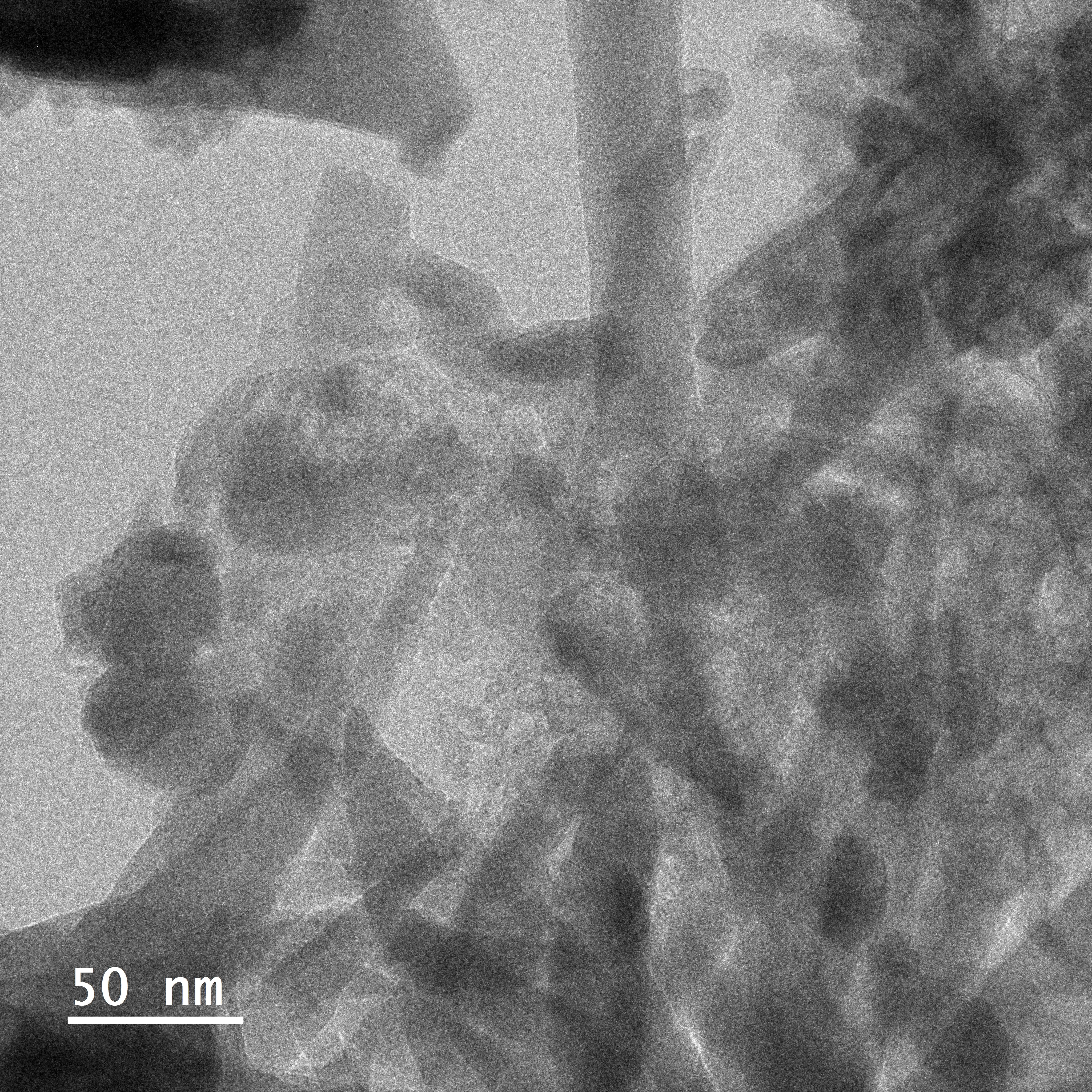

Supplement: Supplementary file 5 — Source Data [file 41467_2024_48564_MOESM5_ESM.zip › Source Data/Supplementary Fig. 16/Supplementary Fig. 16c.jpg]

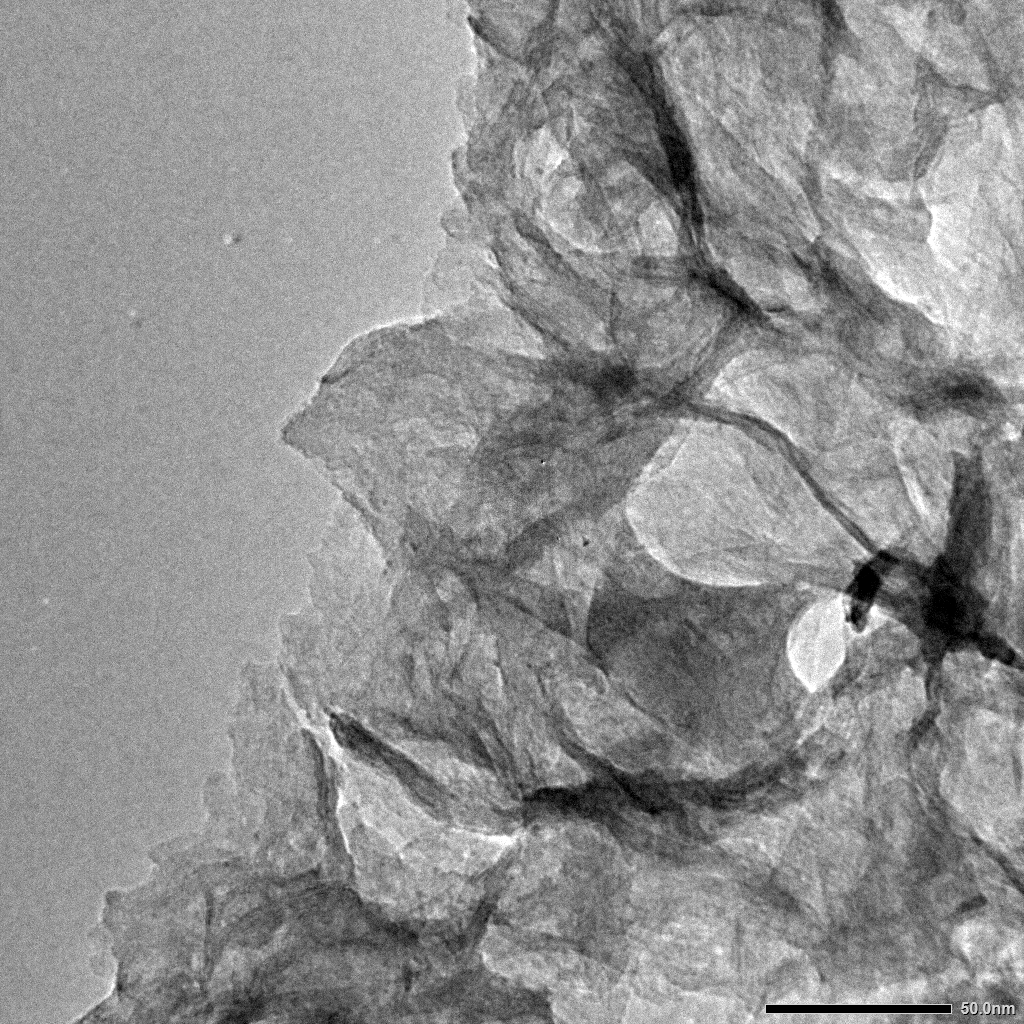

Supplement: Supplementary file 5 — Source Data [file 41467_2024_48564_MOESM5_ESM.zip › Source Data/Supplementary Fig. 16/Supplementary Fig. 16d.bmp]

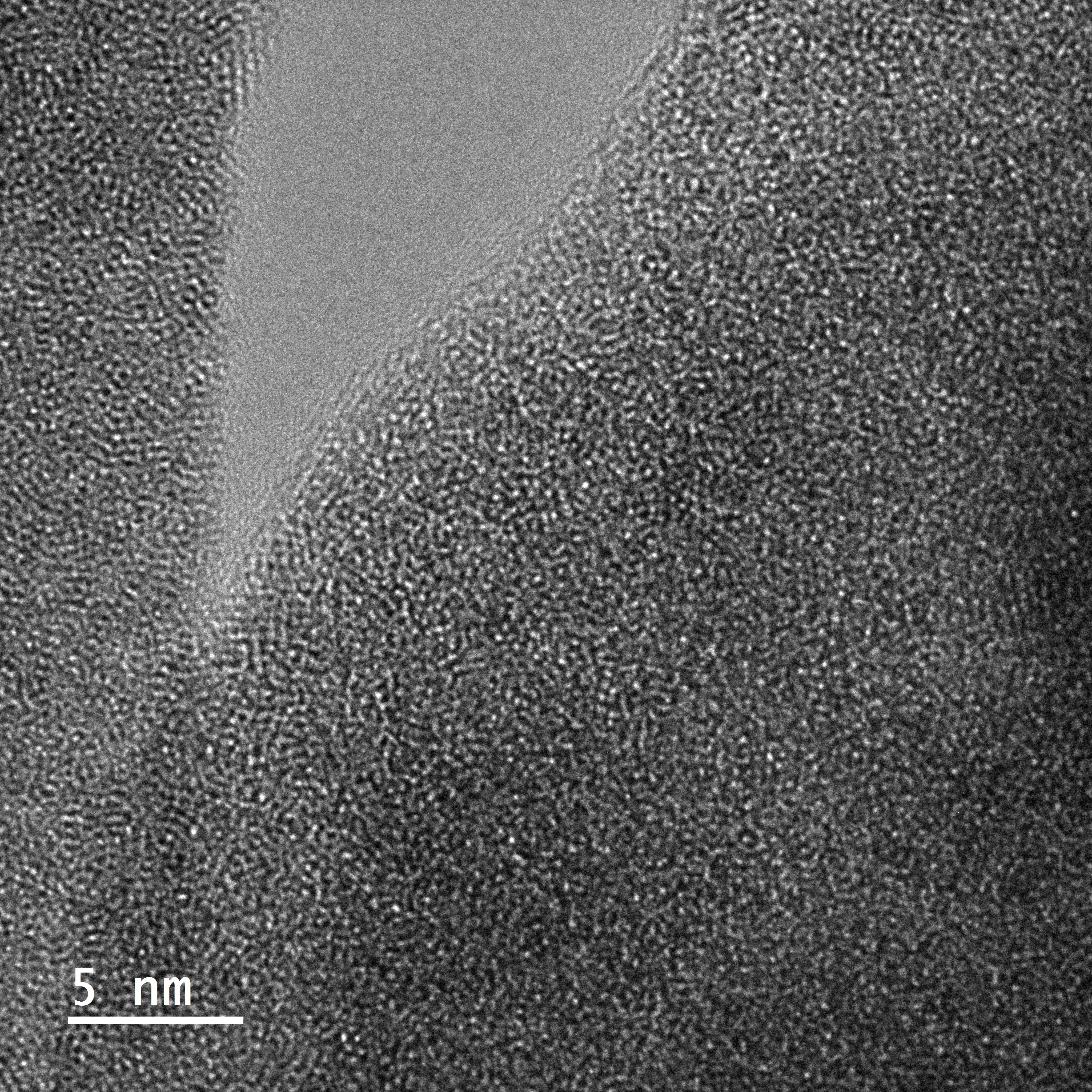

Supplement: Supplementary file 5 — Source Data [file 41467_2024_48564_MOESM5_ESM.zip › Source Data/Supplementary Fig. 16/Supplementary Fig. 16e.jpg]

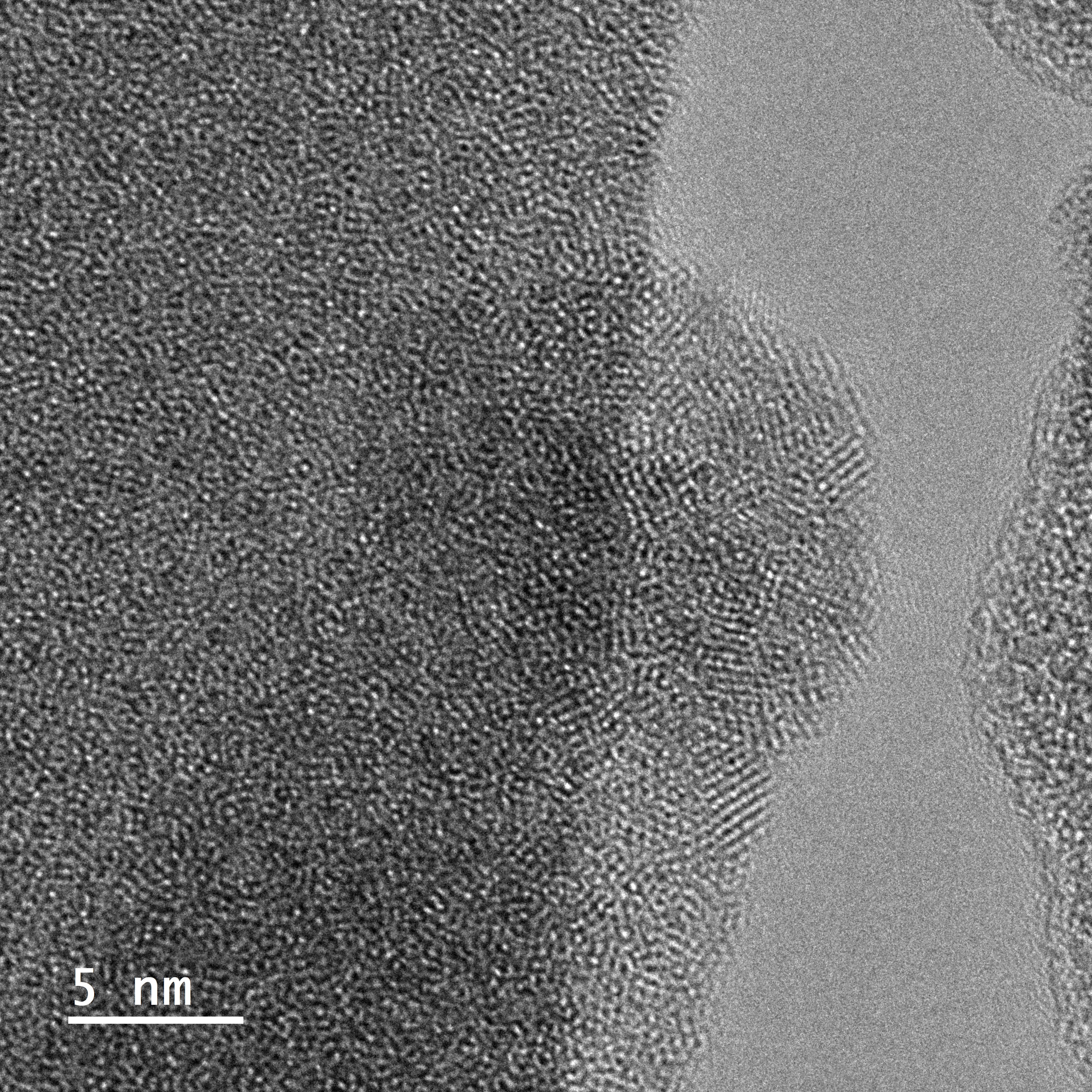

Supplement: Supplementary file 5 — Source Data [file 41467_2024_48564_MOESM5_ESM.zip › Source Data/Supplementary Fig. 16/Supplementary Fig. 16f.jpg]

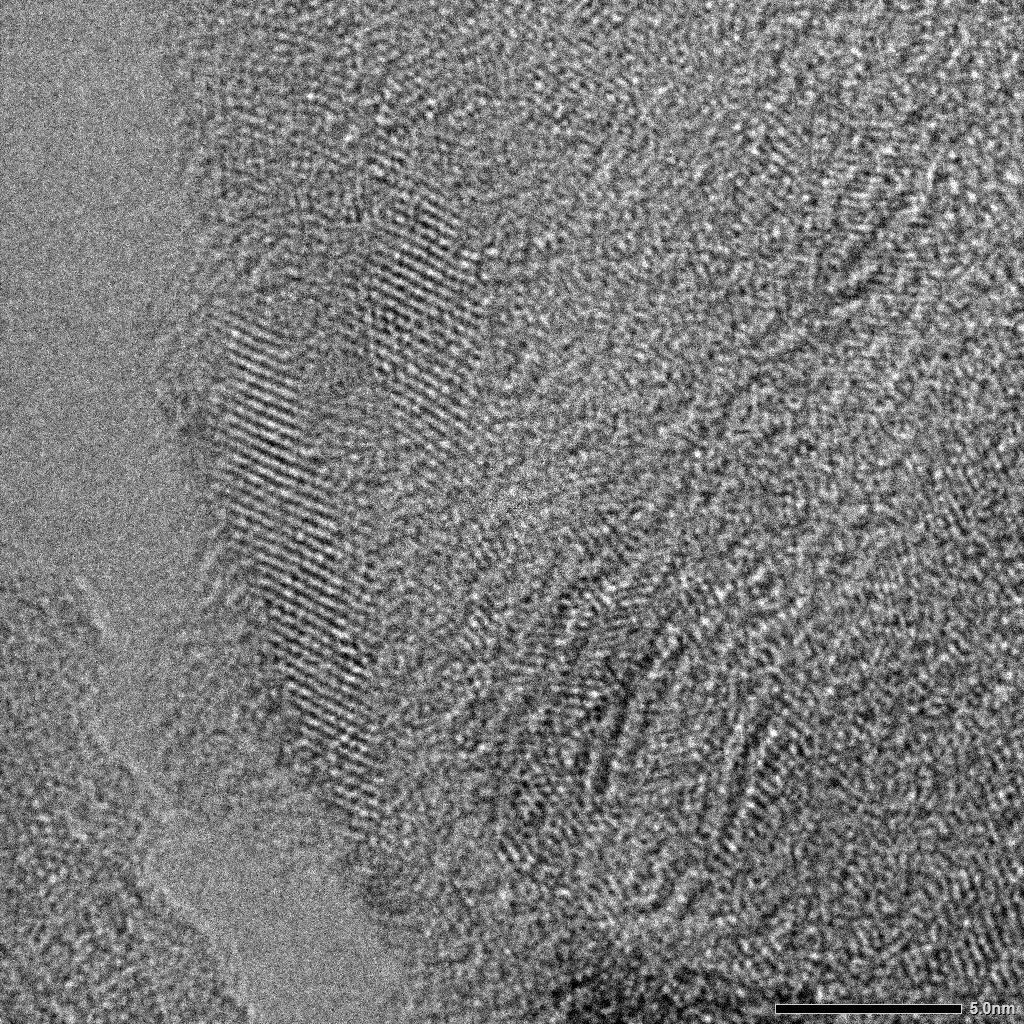

Supplement: Supplementary file 5 — Source Data [file 41467_2024_48564_MOESM5_ESM.zip › Source Data/Supplementary Fig. 16/Supplementary Fig. 16g.bmp]

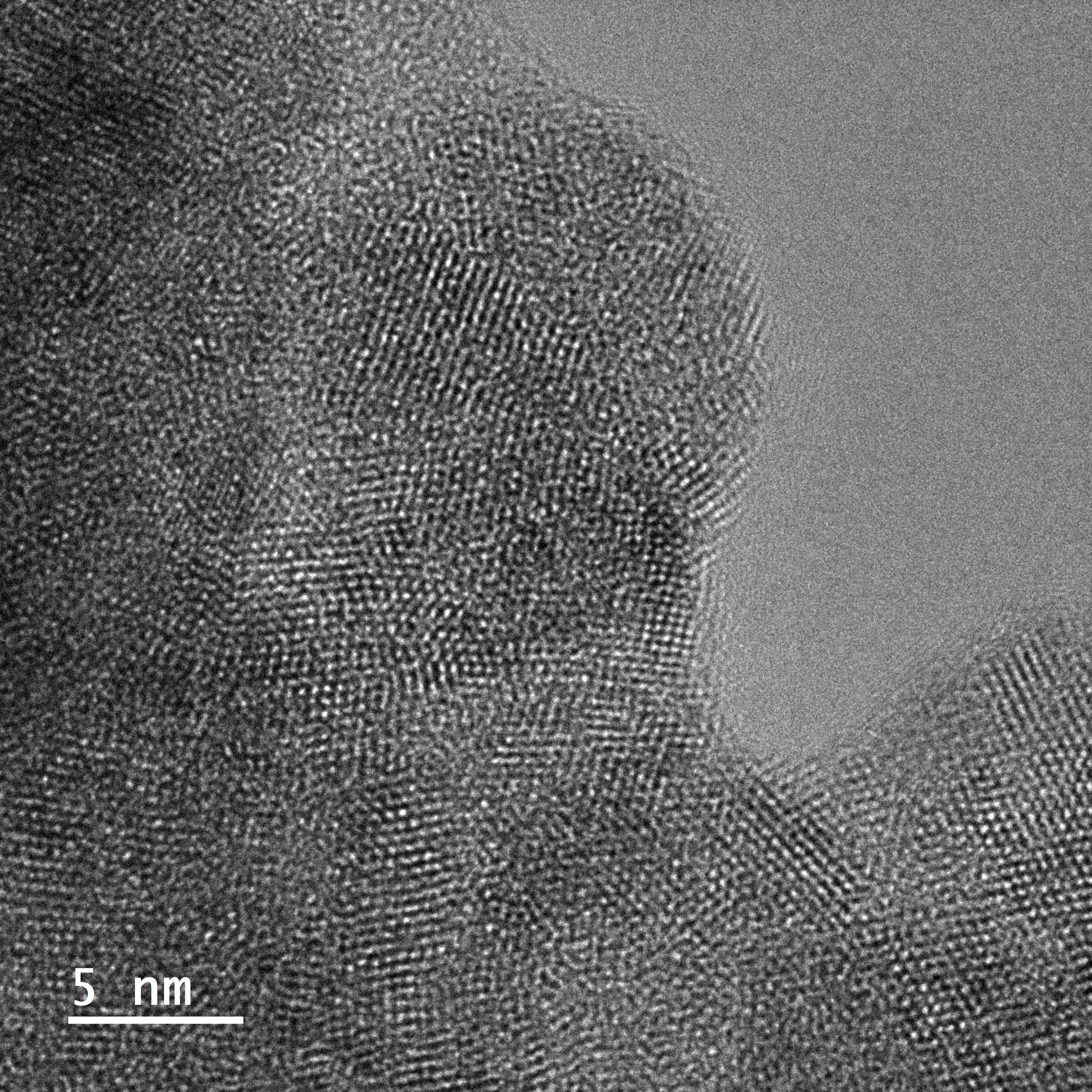

Supplement: Supplementary file 5 — Source Data [file 41467_2024_48564_MOESM5_ESM.zip › Source Data/Supplementary Fig. 16/Supplementary Fig. 16h.jpg]

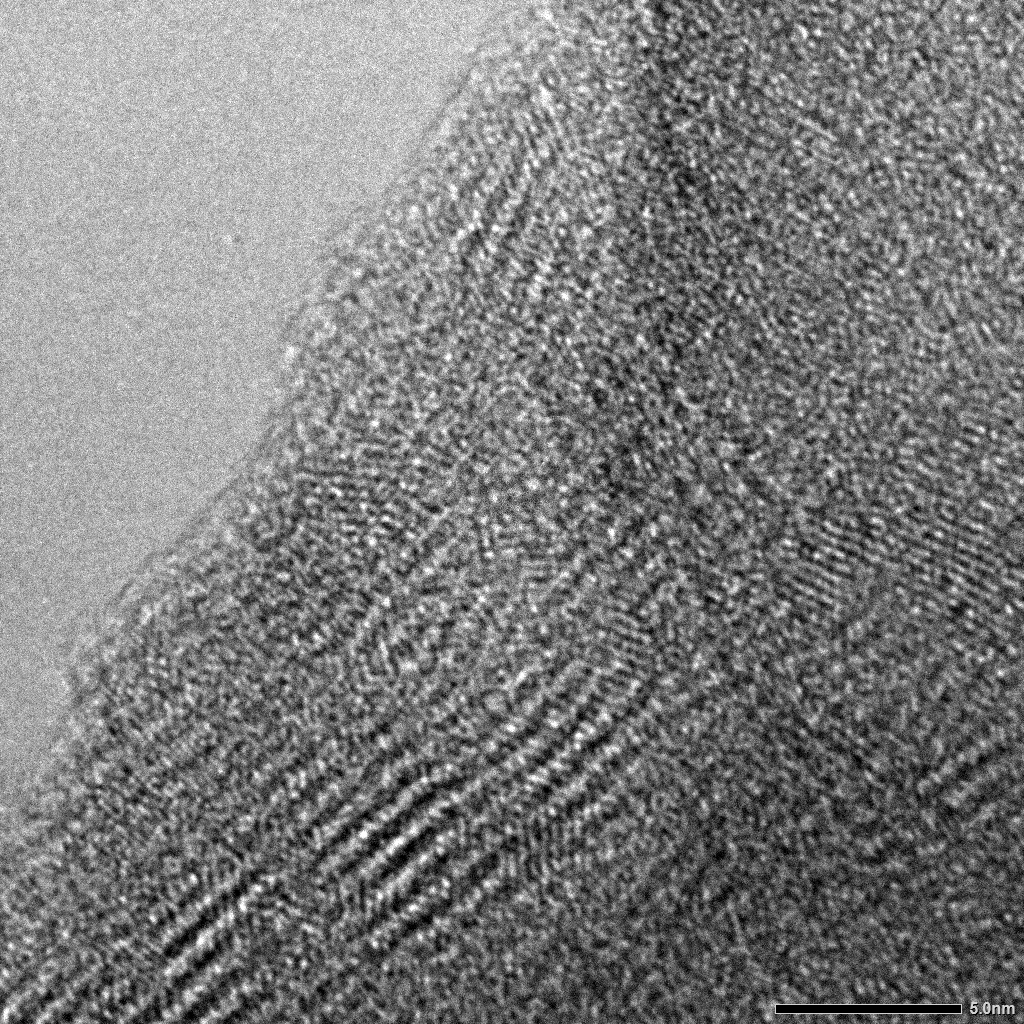

Supplement: Supplementary file 5 — Source Data [file 41467_2024_48564_MOESM5_ESM.zip › Source Data/Supplementary Fig. 17/Ti(OH)PO4 after 1h reaction.bmp]

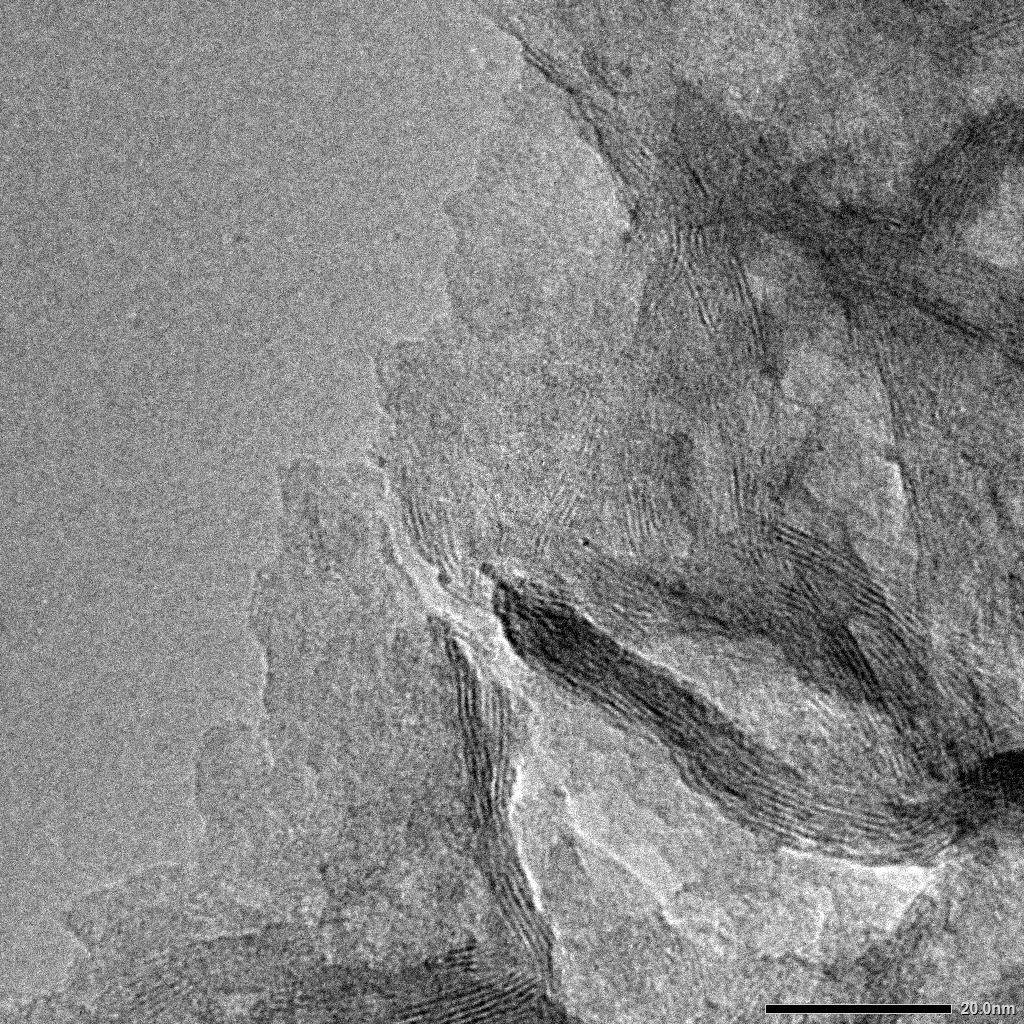

Supplement: Supplementary file 5 — Source Data [file 41467_2024_48564_MOESM5_ESM.zip › Source Data/Supplementary Fig. 17/Ti(OH)PO4 after 7h reaction.bmp]

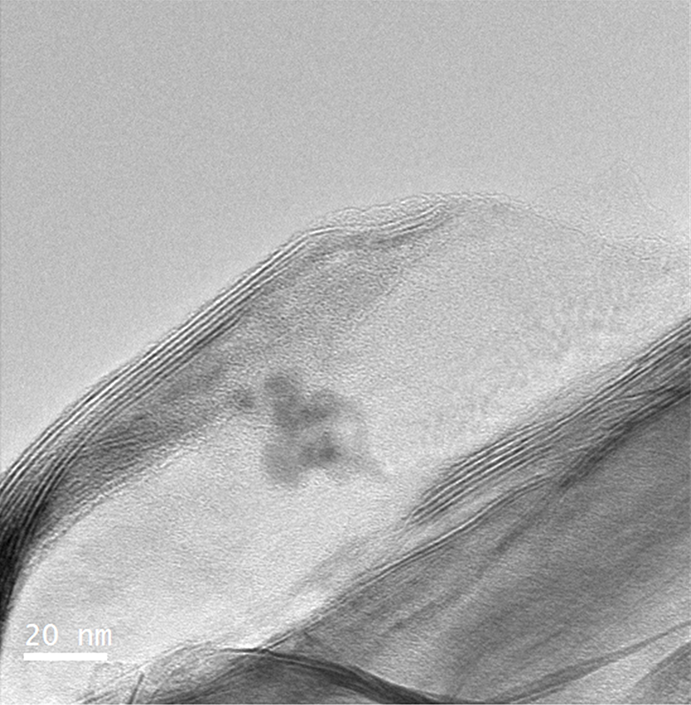

Supplement: Supplementary file 5 — Source Data [file 41467_2024_48564_MOESM5_ESM.zip › Source Data/Supplementary Fig. 2/Supplementary Fig. 2a.bmp]

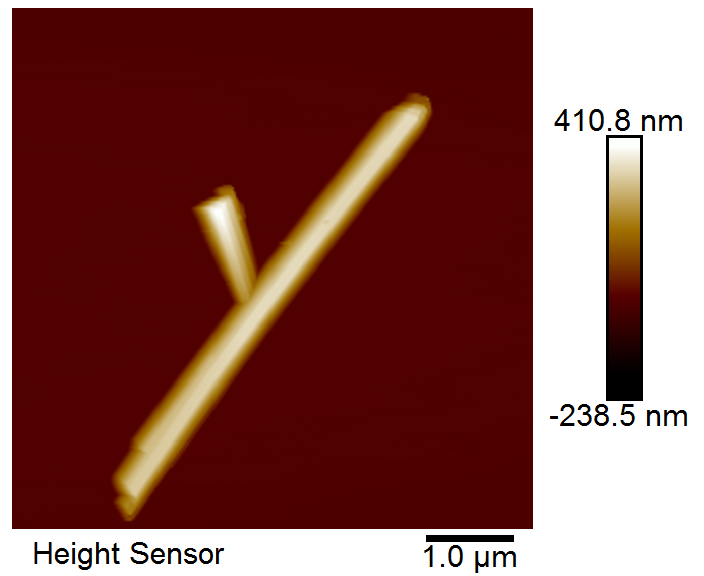

Supplement: Supplementary file 5 — Source Data [file 41467_2024_48564_MOESM5_ESM.zip › Source Data/Supplementary Fig. 4/Supplementary Fig. 4a.tif]

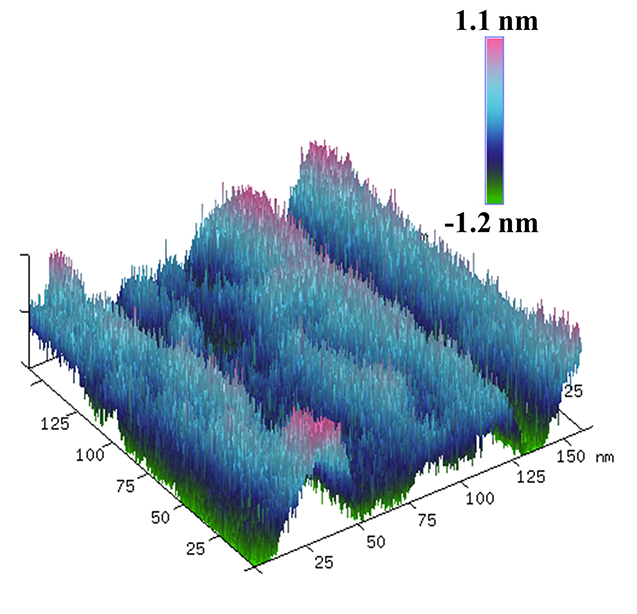

Supplement: Supplementary file 5 — Source Data [file 41467_2024_48564_MOESM5_ESM.zip › Source Data/Supplementary Fig. 4/Supplementary Fig. 4c.bmp]

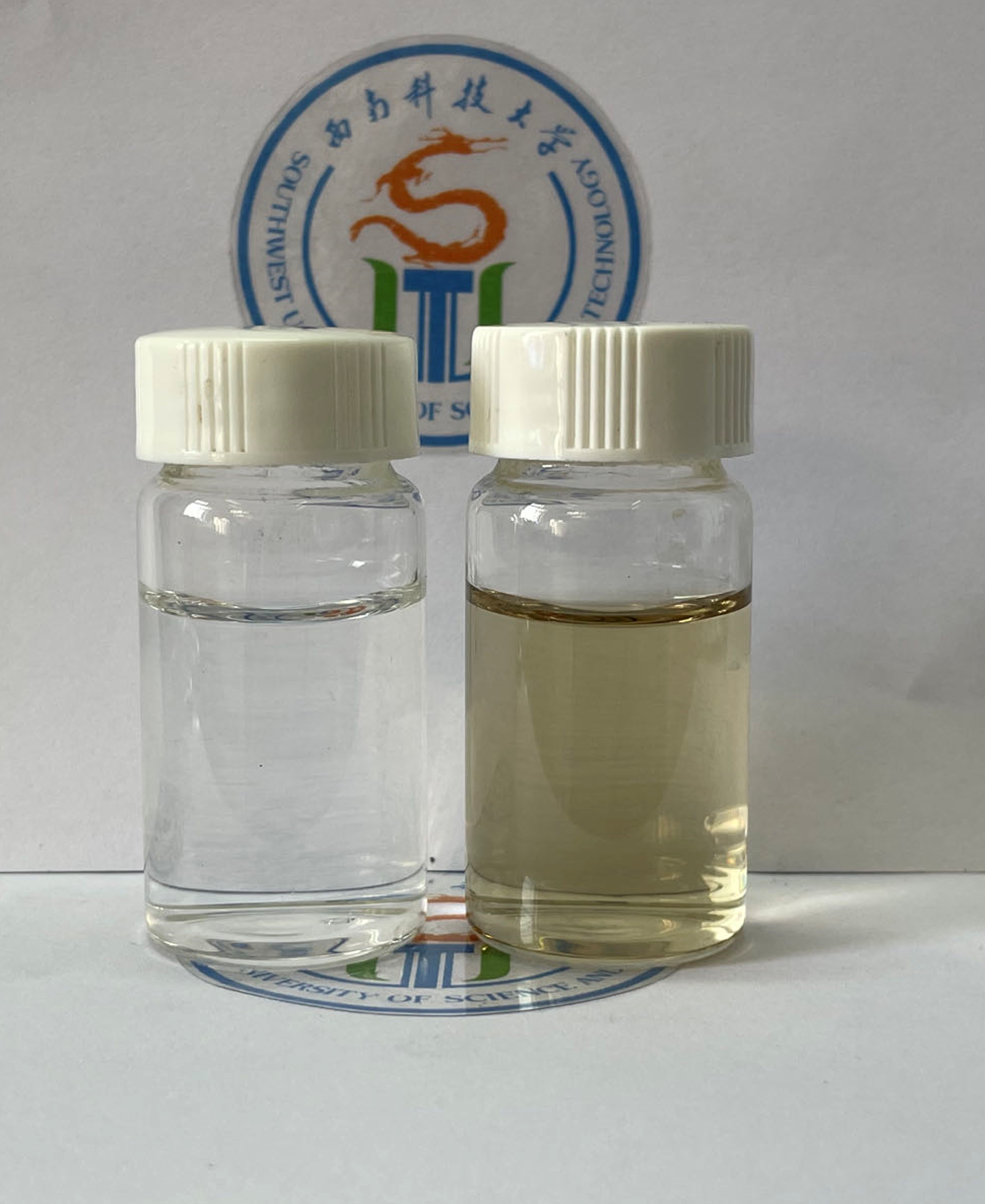

Supplement: Supplementary file 5 — Source Data [file 41467_2024_48564_MOESM5_ESM.zip › Source Data/Supplementary Fig. 9.bmp]
